# Caspase-6-cleaved Tau fails to induce Tau hyperphosphorylation and aggregation, neurodegeneration, glial inflammation, and cognitive deficits

**DOI:** 10.1038/s41419-021-03506-0

**Published:** 2021-03-01

**Authors:** Anastasia Noël, Bénédicte Foveau, Andréa C. LeBlanc

**Affiliations:** 1 Bloomfield Center for Research in Aging, Lady Davis Institute for Medical Research, Jewish General Hospital, Montreal, QC Canada; 2 Department of Neurology and Neurosurgery, McGill University, Montreal, QC Canada; 3 Department of Anatomy and Cell Biology, McGill University, Montreal, QC Canada

**Keywords:** Mechanisms of disease, Cellular neuroscience, Cognitive ageing, Hippocampus, Alzheimer's disease

## Abstract

Active Caspase-6 (Casp6) and Tau cleaved by Casp6 at amino acids 402 (Tau∆D402) and 421 (Tau∆D421) are present in early Alzheimer disease intraneuronal neurofibrillary tangles, which are made primarily of filamentous Tau aggregates. To assess whether Casp6 cleavage of Tau contributes to Tau pathology and Casp6-mediated age-dependent cognitive impairment, we generated transgenic knock-in mouse models that conditionally express full-length human Tau (hTau) 0N4R only (CTO) or together with human Casp6 (hCasp6) (CTC). Region-specific hippocampal and cortical hCasp6 and hTau expression were confirmed with western blot and immunohistochemistry in 2–25-month-old brains. Casp6 activity was confirmed with Tau∆D421 and Tubulin cleaved by Casp6 immunopositivity in 3–25-month-old CTC, but not in CTO, brains. Immunoprecipitated Tau∆D402 was detected in both CTC and CTO brains, but was more abundant in CTC brains. Intraneuronal hippocampal Tau hyperphosphorylation at S202/T205, S422, and T231, and Tau conformational change were absent in both CTC and CTO brains. A slight accumulation of Tau phosphorylated at S396/404 and S202 was observed in Cornu Ammonis 1 (CA1) hippocampal neuron soma of CTC compared to CTO brains. Eighteen-month-old CTC brains showed rare argentophilic deposits that increased by 25 months, whereas CTO brains only displayed them sparsely at 25 months. Tau microtubule binding was equivalent in CTC and CTO hippocampi. Episodic and spatial memory measured with novel object recognition and Barnes maze, respectively, remained normal in 3–25-month-old CTC and CTO mice, in contrast to previously observed impairments in ACL mice expressing equivalent levels of hCasp6 only. Consistently, the CTC and CTO hippocampal CA1 region displayed equivalent dendritic spine density and no glial inflammation. Together, these results reveal that active hCasp6 co-expression with hTau generates Tau cleavage and rare age-dependent argentophilic deposits but fails to induce cognitive deficits, neuroinflammation, and Tau pathology.

## Introduction

Tau is expressed as six isoforms in the human adult brain^1^, is principally located in axons, and promotes microtubules (MT) assembly and stabilization^2^. In Alzheimer disease (AD), Tau aggregates as paired helical filaments-forming neurofibrillary tangles (NFT) that accumulate in neuropil threads and neuritic plaques^3^. NFT initially occur in the trans-entorhinal region, progressively invade the brain through the subiculum to the hippocampus, and then cortical areas^4^. NFT density correlates with cognitive decline^5^, supporting a central role of Tau in AD.

Evidence suggests that caspase-cleaved Tau influences Tau pathogenesis and is involved in cognitive deficits. Caspases cleave human Tau (hTau) at D13 (TauΔD13), D314, D402 (TauΔD402), and D421 (TauΔD421)^6^. In AD brains, TauΔD402 and TauΔD421 are observed in pre-, mature, and ghost NFT, neuropil threads, and neuritic plaques^7–9^. Tau∆D402 immunopositive NFT levels in the entorhinal cortex and the hippocampal Cornu Ammonis 1 (CA1) regions correlate negatively with global cognitive and mini mental state exam scores, and episodic and semantic memory performance^10–12^. Furthermore, cerebrospinal fluid Tau∆D402 levels reflect those in brain and correlate positively with AD severity^13^. Tau∆D421 CA1 levels inversely correlate with mini mental state exam scores^7^, and serum levels separate AD and mild cognitively impaired patients from those with other dementias^14^. Moreover, Tau cleaved at D314 brain levels are increased in mild cognitively impaired and AD individuals^15,16^. TauΔD421 induces mitochondrial dysfunction and neurite loss in neuronal cultures^17–22^ and increases in vitro Tau polymerization^23^ and aggregation^7^. In TauP301S and TauP301L transgenic mice, TauΔD421 is present in Tau aggregates^24–26^. Caspase activation precedes and induces tangle formation in the Tg4510 mouse expressing TauP301L^27^. Tau pretangle pathology is observed in transgenic mice expressing hTau_1-421_ (TauC3)^28^ and in mice with inducible hTau_151-421_ expression (TAU62)^29^. Intracellular Tau aggregation, induced with AD brain high molecular weight protein fraction, is decreased after Tau∆D421 immunodepletion^30^, suggesting that Tau∆D421 might participate in Tau pathology spreading. Moreover, adeno-associated viral (AAV)-directed expression of hTau_1-421_ in wild-type mice results in Tau oligomers, intracellular hyperphosphorylated misfolded Tau, endogenous Tau recruitment to aggregates, microgliosis, and neurodegeneration^27,31^. Furthermore, TauC3 and TAU62 mice show memory impairments and synaptic alterations^28,29^. In contrast, transgenic mice expressing uncleavable D421 endogenous murine Tau exhibit long-term potentiation and cognitive deficits^32^.

TauΔD421 is generated by Caspase-3 (Casp3), Casp6 (Casp6), and Caspase-7 (refs. ^7,23,33^), whereas Tau∆D402 is generated by Casp6 (ref. ^8^). Active Casp3 and Caspase-7 are sparse in AD brains^9,34^. However, active Casp6 and Tau∆D402 are present in NFT, neuritic plaques, and neuropil threads in sporadic and familial AD, mild cognitively impaired individuals, and some non-cognitively impaired aged brains^8,10,35^, but absent in brains without AD pathologies^10,11^. In non-cognitively impaired individuals, active Casp6 is observed only in the entorhinal cortex and the CA1 region, the first areas to present with NFT according to Braak staging^4^, and its levels inversely correlate with cognitive and episodic memory scores^10,11^. Transgenic expression or injection of active human Casp6 (hCasp6) in mouse CA1 neurons is sufficient to induce age-dependent cognitive impairment, synaptic transmission deficits, neuroinflammation, and neurodegeneration, but does not generate NFT^12,36,37^. Active Casp6 causes axonal degeneration in neuron cultures^38–40^ and increases amyloid beta production^41,42^. Consistent with its role in axonal degeneration, Casp6 cleaves numerous cytoskeleton or cytoskeleton-associated proteins including Tau, α-tubulin, and post-synaptic density proteins^8,43^. Furthermore, Casp6 impairs proteasomal degradation of misfolded proteins by cleaving p97 (ref. ^44^). Together, these data suggest that the active Casp6 in AD brains could be a major contributor to cognitive decline.

To assess whether Casp6-cleaved Tau is toxic or produces Tau abnormalities, we generated new transgenic mice that conditionally express full-length hTau alone (CTO) or with hCasp6 (CTC) in hippocampal CA1 and cortical neurons. Caspase-cleaved Tau was observed by 3 months of age, but significant Tau hyperphosphorylation, altered Tau binding to MT, or NFT pathology were not detected even in 25-month-old brains. Rare argentophilic deposits were observed in old mice. Surprisingly, CTC and CTO mice displayed normal memory and dendritic spine density, and did not show signs of neuroinflammation, indicating the absence of neurodegeneration. Together, these data suggest that Casp6-cleaved Tau is not toxic to CA1 and cortical neurons and is insufficient to generate obvious Tau pathology.

## Results

### Co-expression of hTau with hCasp6 generates cleaved Tau

#### Human Casp6 is specifically observed in the CTC KI/Cre hippocampus and cortex

**Fig. 1:**
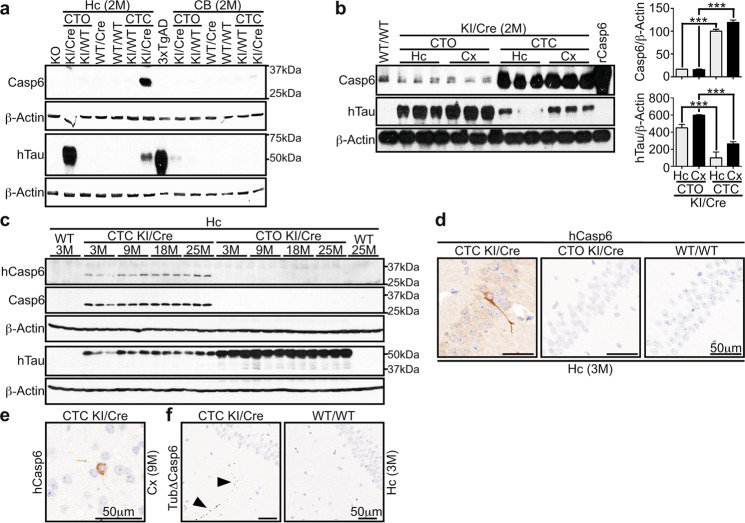
Specific co-expression of active hCasp6 with hTau in the cortex and hippocampus of CTC KI/Cre mice. Western blot with 15 μg total proteins from (**a, b**) 2 month (M) old and (**c**) 3–25-month-old CTO KI/Cre, CTO KI/WT, WT/Cre, WT/WT, CTC KI/WT, or CTC KI/Cre hippocampus (Hc), cerebellum (CB), or cortex (Cx) with anti-Casp6, anti-hTau, anti-hCasp6, and anti-β-Actin antibodies. In **a**, proteins from Casp6 KO hippocampus or Tau KO brainstem (KO) were used as negative controls for Casp6 and Tau analyses, respectively. Cx proteins from 3xTgAD were used as positive control for hTau. In **b**, the histograms represent densitometric analyses of the western blot shown. Data are expressed as ratios of Casp6 or hTau over β-Actin, relative to CTC KI/Cre hippocampus (*n* = 3/genotype/structure). One-way ANOVA followed by Bonferroni’s post-hoc test was performed. ***p < 0.001. **d-f** Representative micrographs from *n* = 5/genotype/structure/age/antibody for 3 M hippocampus (**d**) and 9 M cortex (**e**) with anti-hCasp6, and 3 M hippocampus with anti-Tub∆Casp6 (**f**).

Transgenic animals with knocked-in (KI) hTau or hTau-hCasp6 cDNA were crossed with mice expressing Cre recombinase (Cre) under the calcium/calmodulin-dependent protein kinase II alpha (CaMKIIα) promoter (Supplementary Fig. 1a). Mice carrying the KI and Cre transgenes (KI/Cre) were compared to wild-type (WT/WT), KI transgene in the absence of Cre (KI/WT), or Cre only (WT/Cre) littermates. Compared to WT/WT, WT/Cre, or CTC KI/WT control animals, CTC KI/Cre mice showed increased Casp6 protein levels in the hippocampus and cortex but not cerebellum (Fig. 1a and b) or peripheral tissues (Supplementary Fig. 1b), indicating region-specific Casp6 overexpression in CTC KI/Cre mice. The overexpressed Casp6 was confirmed to be human with the LS-B477 antibody (Fig. 1c). Multiplex PCR analyses showed specific STOP-cassette excision in the hippocampus and cortex of CTC KI/Cre mice, but not in other tissues or genotypes (Supplementary Fig. 1c), confirming specific Casp6 overexpression under the CaMKIIα-Cre action.

Casp6 protein (Fig. 1b) and mRNA (Supplementary Fig. 1d) levels were the same in CTC KI/Cre hippocampus and cortex, indicating similar expression of hCasp6 cDNA in these two regions. Hippocampal Casp6 protein levels were the same in CTC and ACL KI/Cre mice (Supplementary Fig. 1e), indicating that ACL KI/Cre were appropriate controls for hCasp6 expression alone.

Casp6 protein overexpression was not observed in CTO brains (Fig. 1a and b) and peripheral tissues (Supplementary Fig. 1b). Similarly, hCasp6 mRNA was not detected in the CTO KI/WT and KI/Cre hippocampus and cortex (Supplementary Fig. 1d), confirming the absence of hCasp6 in CTO mice.

Hippocampal hCasp6 was detected in CTC KI/Cre throughout aging (Fig. 1c). Both hCasp6 and total Casp6 protein levels increased with aging (Supplementary Fig. 1f). Hippocampal hCasp6 levels were ~2.3 fold higher at 25 months compared to 3 months of age (*p* = 0.0351). Brain sections from 3 to 25-month-old mice showed hCasp6 immunopositivity in the cell soma and neurites of CTC KI/Cre CA1 neurons at all ages (Fig. 1d and Supplementary Fig. 2a) and in the cortex at 9, 18, and 25 months (Fig. 1e and Supplementary Fig. 2b).

Anti-his-tag immunopositivity in CTC KI/Cre sections was more abundant than hCasp6 immunostaining (Supplementary Fig. 2a and c). This may be related to different avidity and affinity of the antibodies or could suggest that hCasp6 underwent auto-processing; the anti-hCasp6 epitope being located in the hCasp6 linker domain. Tubulin cleaved by Casp6 (TubΔCasp6) immunopositivity confirmed Casp6 activity in CTC KI/Cre hippocampus as early as 3 months of age (Fig. 1f), and throughout aging (Supplementary Fig. 2d). TubΔCasp6 was detected in CA1 pyramidal neurons and in punctate structures in the stratum radiatum, stratum oriens and stratum lacunosum moleculare (Fig. 1f and Supplementary Fig. 2d), similar to the pattern observed with anti-hCasp6 (Supplementary Fig. 2a). TubΔCasp6 was not detected in CTO KI/Cre and control genotypes (Fig. 1f and Supplementary Fig. 2d), confirming that active hCasp6 is specifically expressed in the CTC KI/Cre hippocampus and cortex.

#### Human Tau is expressed in the CTO and CTC KI/Cre cortex and hippocampus

Human Tau was detected in hippocampal and cortical lysates from CTO and CTC KI/Cre, but not from control genotypes (Fig. 1a and b) or peripheral tissues (Supplementary Fig. 3a). The STOP cassette was deleted in CTO KI/Cre hippocampus and cortex, but not in other tissues or genotypes (Supplementary Fig. 3b), confirming Cre-mediated hTau expression in KI/Cre brains.

Human Tau protein levels were equivalent in the hippocampus of 3–25-month-old KI/Cre mice (Fig. 1c and Supplementary Fig. 3c). However, hTau levels were higher in CTO KI/Cre compared to CTC KI/Cre at all ages tested (Fig. 1a–c and Supplementary Fig. 3c). Furthermore, hTau protein levels were similar in the hippocampus and cortex of either CTO or CTC KI/Cre mice (Fig. 1b). Human Tau mRNA levels were increased by 3.5 fold in CTO KI/Cre cortex and hippocampus compared to CTC KI/Cre tissues at 2 months of age (Supplementary Fig. 3d), suggesting that higher hTau protein levels in CTO KI/Cre were likely due to higher amount of mRNA.

Together, these data confirm conditional genotype- and tissue-specific hCasp6 and hTau expression in the CTO and CTC models.

#### Cleaved Tau is detected in hCasp6-hTau expressing brains

**Fig. 2:**
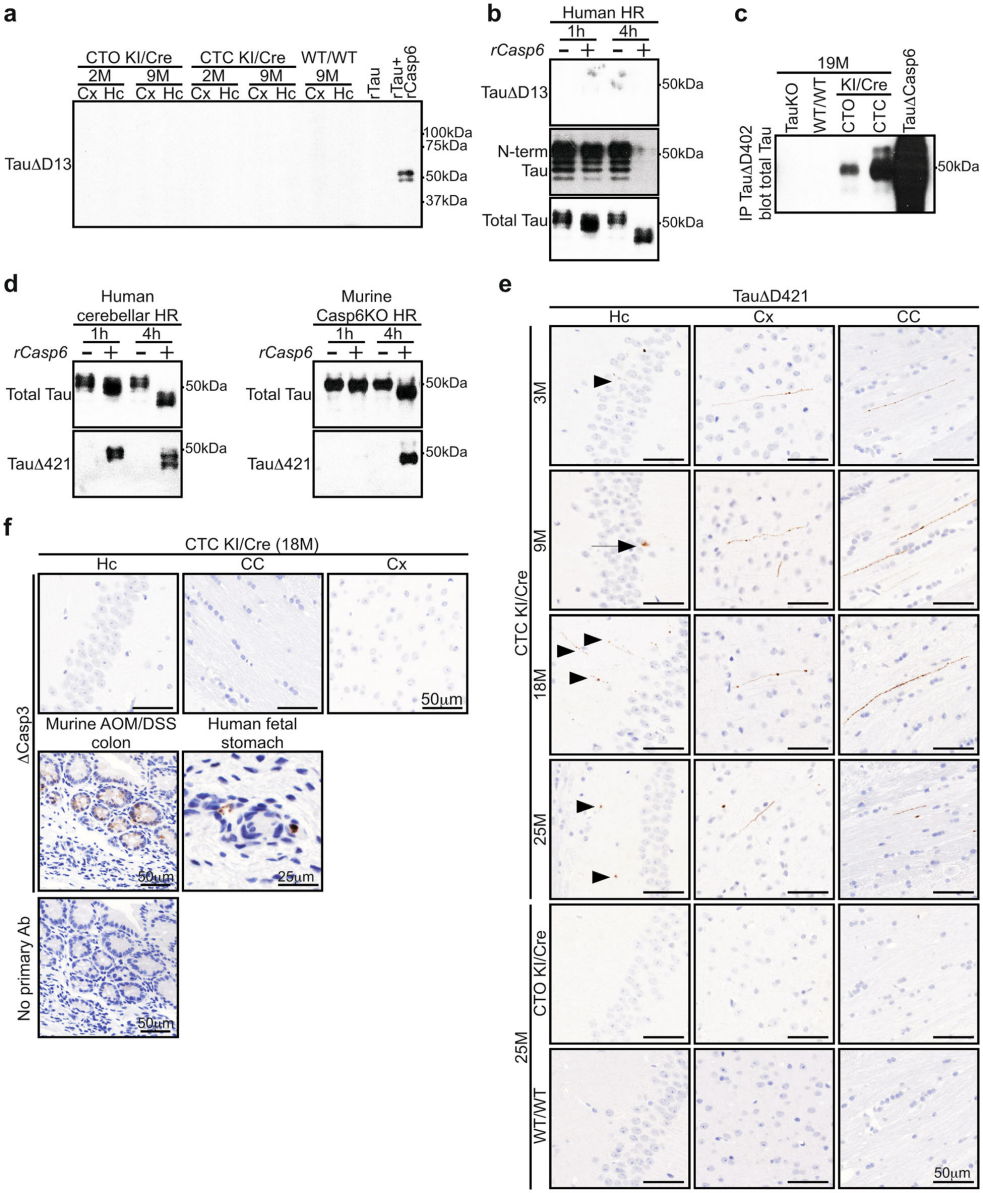
Cleaved Tau is observed in KI/Cre brains. **a** Western blot analyses of 20 μg total proteins from 2- and 9-month-old CTO KI/Cre, CTC KI/Cre, and WT/WT hippocampal (Hc) and cortical (Cx) proteins with anti-TauΔD13. **b** Western blot analyses with anti-TauΔD13, anti-hTau (N-term Tau), and anti-total Tau antibodies of heat-resistant proteins (HR) from human cerebellum lysate incubated with recombinant human active Casp6p20p10 (rCasp6) (+) or vehicle (−) for 1 h or 4 h. **c** TauΔD402 fragments were immunoprecipitated from 19-month-old Tau KO, WT/WT, CTO, and CTC KI/Cre brain lysates with the anti-TauΔD402 antibody, and analyzed by western blotting with anti-total Tau antibody. Recombinant hTau digested with rCasp6 (TauΔCasp6) was used as positive control. **d** Western blot analyses with anti-total Tau and anti-TauΔD421 antibodies of HR from human cerebellum or Casp6 KO mouse brainstem lysate incubated with rCasp6 (+) or vehicle (−) for 1 h or 4 h. **e, f** Representative micrographs of **e** CTC KI/Cre aged from 3 to 25 months (3-month-old: *n* = 5, 9-month-old: *n* = 5, 18-month-old: *n* = 4, 25-month-old: *n* = 3), and 25-month-old CTO KI/Cre (*n* = 4) and WT/WT (*n* = 3), or **f** 18-month-old CTC KI/Cre (*n* = 4) hippocampus (Hc), corpus callosum (CC), and cortex (Cx) tissue sections immunostained with **e** anti-TauΔD421 or **f** anti-∆Casp3 antibody. Sections from AOM-DSS-treated mouse colon and human fetal stomach were used as controls.

Casp6 cleaves Tau at D13, D402, and D421. TauΔD13 was assessed with the Tau-C6g antibody whose epitope is absent in murine Tau. Our results indicated that this antibody was not specific under native conditions. Tau-C6g immunopositivity was observed in brain sections from 9-month-old WT/WT, CTC and CTO KI/Cre (Supplementary Fig. 4a). Adsorption on recombinant hTau (rTau) digested by recombinant active hCasp6 (rCasp6) did not completely eliminate Tau-C6g immunopositivity in human AD brain (Supplementary Fig. 4b). In contrast, under denaturant western blot conditions, Tau-C6g detected TauΔD13 in rTau incubated with rCasp6 (Fig. 2a and Supplementary Fig. 4c), but not in cortical and hippocampal proteins from 2- and 9-month-old CTO and CTC KI/Cre (Fig. 2a). However, Casp6 cleaved the N-terminal 9-18 amino acids of hTau because this epitope disappeared after a 4 h incubation with Casp6 in human heat-resistant proteins (Fig. 2b). Since Tau-C6g recognized Casp6-cleaved rTau (Fig. 2a), post-translational modifications might have masked the epitope, therefore not allowing us to conclude whether TauΔD13 was present in CTC brains.

Brain sections from 3 to 25-month-old KI/Cre were negative for anti-TauΔD402 despite abundant immunopositivity in human AD brains (Supplementary Fig. 4d). However, TauΔD402 was immunoprecipitated in CTC KI/Cre brain lysates, but not in Tau Knock-Out (KO) or WT/WT brains (Fig. 2c). Unexpectedly, Tau∆D402 was also immunoprecipitated from CTO mice brains, although at much lower levels than in CTC brains, suggesting that Tau overexpression alone promoted TauΔD402. Hippocampal sections were stained with anti-ΔCasp6 to determine whether Tau overexpression in CTO mouse brains activated Casp6 (Supplementary Fig. 4e). ΔCasp6 was detected in human AD and CTC KI/Cre, but not CTO sections, suggesting that active Casp6 in CTO KI/Cre was absent or below the detection limit, or that TauΔD402 was generated by another protease.

To confirm hCasp6 cleavage of Tau at D421, human or Casp6 KO mouse heat-resistant proteins were incubated with or without rCasp6 (Fig. 2d). TauΔD421 was detected only in samples treated with rCasp6. Casp3, however, was undetected in heat-resistant proteins, despite being easily detected in unheated lysate, heat-treated pellet, and normal and tumor colon tissues, as expected (Supplementary Fig. 4f). Furthermore, TauΔD421 was generated when rTau was incubated with rCasp6 (Supplementary Fig. 4g), confirming direct Tau cleavage at D421 by hCasp6. TauΔD421 was observed mainly in the corpus callosum, and to a lesser extent in the hippocampus and cortex of CTC KI/Cre brain sections, but not in other genotypes (Fig. 2e and Supplementary Fig. 4h), thus indicating specific Tau cleavage at D421 in CTC KI/Cre brains. At all ages tested, TauΔD421 immunopositivity appeared as punctate structures in neuritic cytoplasm of the hippocampus, corpus callosum and cortex (Fig. 2e). In addition, at 9 months TauΔD421 was occasionally observed in CA1 pyramidal neuron soma.

Despite active Casp3 immunopositivity in positive controls, active Casp3 was not detected in the CTC KI/Cre hippocampus, corpus callosum and cortex (Fig. 2f). Together, these results suggest that Casp6 generates TauΔD402/D421 in the CTC KI/Cre brains.

### CTC KI/Cre mice are cognitively normal

#### CTC KI/Cre mice do not display locomotor impairments

To assess whether the presence of cleaved Tau was associated with hippocampal and cortical dysfunction, 3–25-month-old mice were submitted to cognitive tests. Animals first underwent an openfield session to ensure normal locomotor function (Supplementary Fig. 5). At each age, the distance travelled (Supplementary Fig. 5a), total number of entries in each openfield zone (Supplementary Fig. 5b), and percentage of time moving (Supplementary Fig. 5c) of transgenic animals were similar to those of WT/WT mice, indicating normal overall activity and motor function. CTC KI/Cre mice explored the same percentage of cells (Supplementary Fig. 5d) and spent the same amount of time in the periphery (Supplementary Fig. 5e) than control mice of the same age. Therefore, CTC KI/Cre mice did not display locomotor defects or anxiety at any age.

#### CTC KI/Cre mice show normal episodic and spatial memory

**Fig. 3:**
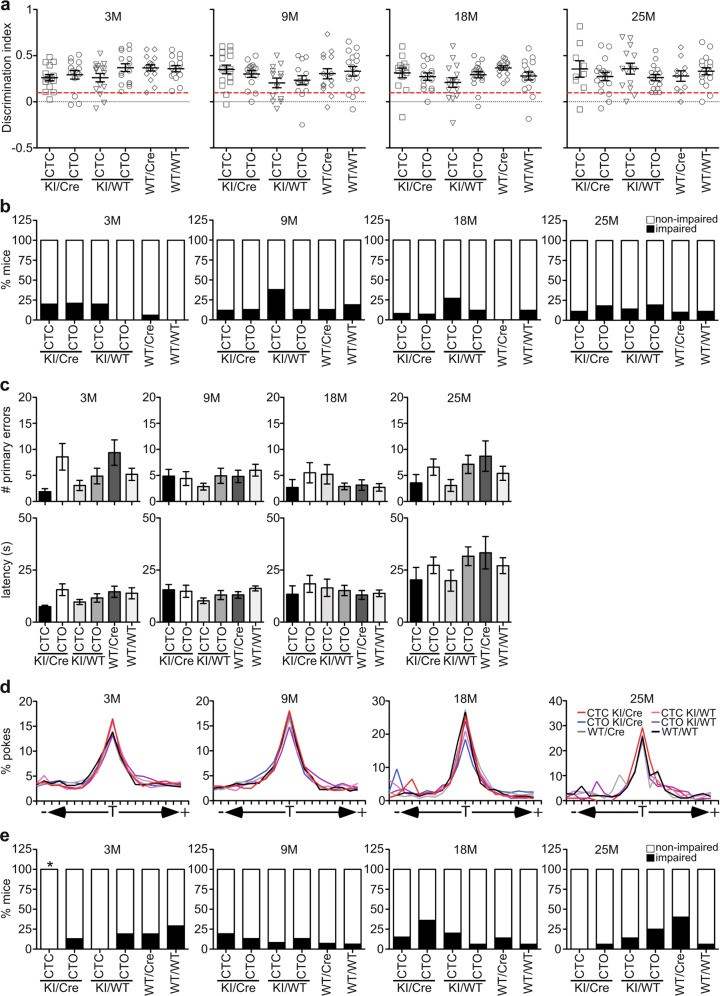
CTC KI/Cre mice are not cognitively impaired. **a, b** NOR and **c–e** Barnes maze analyses of 3–25-month-old CTC KI/Cre (3 M: *n* = 15, 9 M: *n* = 16, 18 M: *n* = 13, 25 M: *n* = 9 for NOR and *n* = 7 for Barnes maze), CTO KI/Cre (3 M: *n* = 14 for NOR and *n* = 15 for Barnes maze, 9 M: *n* = 15, 18 M: *n* = 14, 25 M: *n* = 17), CTC KI/WT (3 M: *n* = 15, 9 M: *n* = 13, 18 M: *n* = 15, 25 M: *n* = 14), CTO KI/WT (3 M: *n* = 16, 9 M: *n* = 15, 18 M: *n* = 17, 25 M: *n* = 16), WT/Cre (3 M: *n* = 16, 9 M: *n* = 15, 18 M: *n* = 14, 25 M: *n* = 10), and WT/WT (3 M: *n* = 15 for NOR and *n* = 14 for Barnes maze, 9 M: *n* = 16, 18 M: *n* = 17, 25 M: *n* = 18 for NOR and *n* = 17 for Barnes maze). **a** Discrimination NOR index. Red line: cut-off for impaired mice (discrimination index = 0.1). **b** Percentage of NOR impaired mice. **c** Number of primary errors and latency to the target, and **d** percentage of pokes made to each hole during the Barnes maze probe test. T target hole. **e** Percentage of Barnes maze impaired mice. *p < 0.05 vs 3-month-old WT/WT, Fisher’s exact test. **a, c** Data represent the mean ± SEM. Statistical evaluations were done with One-way ANOVA followed by a Bonferroni’s post-hoc analysis.

CTC and CTO KI/Cre mice performed normally in the novel object recognition test, significantly spending more time touching the novel object than the familiar object at 3, 9, 18, and 25 months of age (Fig. 3a and Supplementary Fig. 6a). The discrimination index was equivalent among the six genotypes groups of the same age (Fig. 3a). At 3 months, the percentage of impaired CTC KI/Cre and KI/WT, and CTO KI/Cre mice seemed higher than WT/WT (Fig. 3b); however, there was no significant difference between these genotypes (Supplementary Table 1). There was also no interaction between age and genotype on discrimination index and percentage of impaired mice. Together, these results indicate normal episodic memory in KI/Cre animals.

Spatial memory was evaluated with the Barnes maze test. The number of primary errors and the primary latency to find the escape hole during learning acquisition significantly decreased over time and to the same extent for the six genotypes at all ages (Supplementary Fig. 6b), indicating effective learning of the task. During the probe test, all genotypes performed equally well. No significant differences were observed in the number of primary errors and the latency to reach the target between KI/Cre mice and control littermates (Fig. 3c), and all genotypes made more pokes to the target compared to the other holes at all ages tested (Fig. 3d), confirming memory recall. Further analysis of individual performance during the probe test showed that CTC KI/Cre performed better than WT/WT mice at 3 months of age (*p* = 0.0421) but not after (Fig. 3e and Supplementary Table 2). Consistent with previous observation of poor spatial memory in old WT/Cre mice^12^, more WT/Cre animals were impaired than mice with other genotypes at 25 months of age.

Together, these data indicate that neither CTC nor CTO KI/Cre mice are impaired in episodic and spatial memory.

### Dendritic spine density is maintained in the old CTC KI/Cre hippocampus

**Fig. 4:**
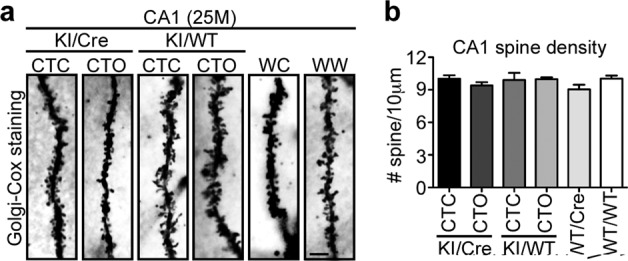
Dendritic spine density is maintained in the hippocampus of old CTC KI/Cre mice. **a** Representative micrographs of Golgi-cox staining and **b** quantification of dendritic spine density in hippocampal CA1 sections from 25-month-old CTC KI/Cre (*n* = 3), CTO KI/Cre (*n* = 5), CTC KI/WT (*n* = 4), CTO KI/WT (*n* = 4), WT/Cre (*n* = 5), and WT/WT (*n* = 4). WC: WT/Cre, WW: WT/WT. Data represent the mean ± SEM. One-way ANOVA followed by Bonferroni’s post-hoc.

Dendritic spines loss is a feature of Tau transgenic mice. Therefore, 25-month-old brains were evaluated by Golgi-Cox impregnation (Fig. 4a, b). CA1 dendritic spine density was the same in CTC and CTO KI/Cre, and their control genotypes, which is consistent with the absence of cognitive deficits in KI/Cre mice.

### Neuroinflammation is absent in old CTC KI/Cre mice

**Fig. 5:**
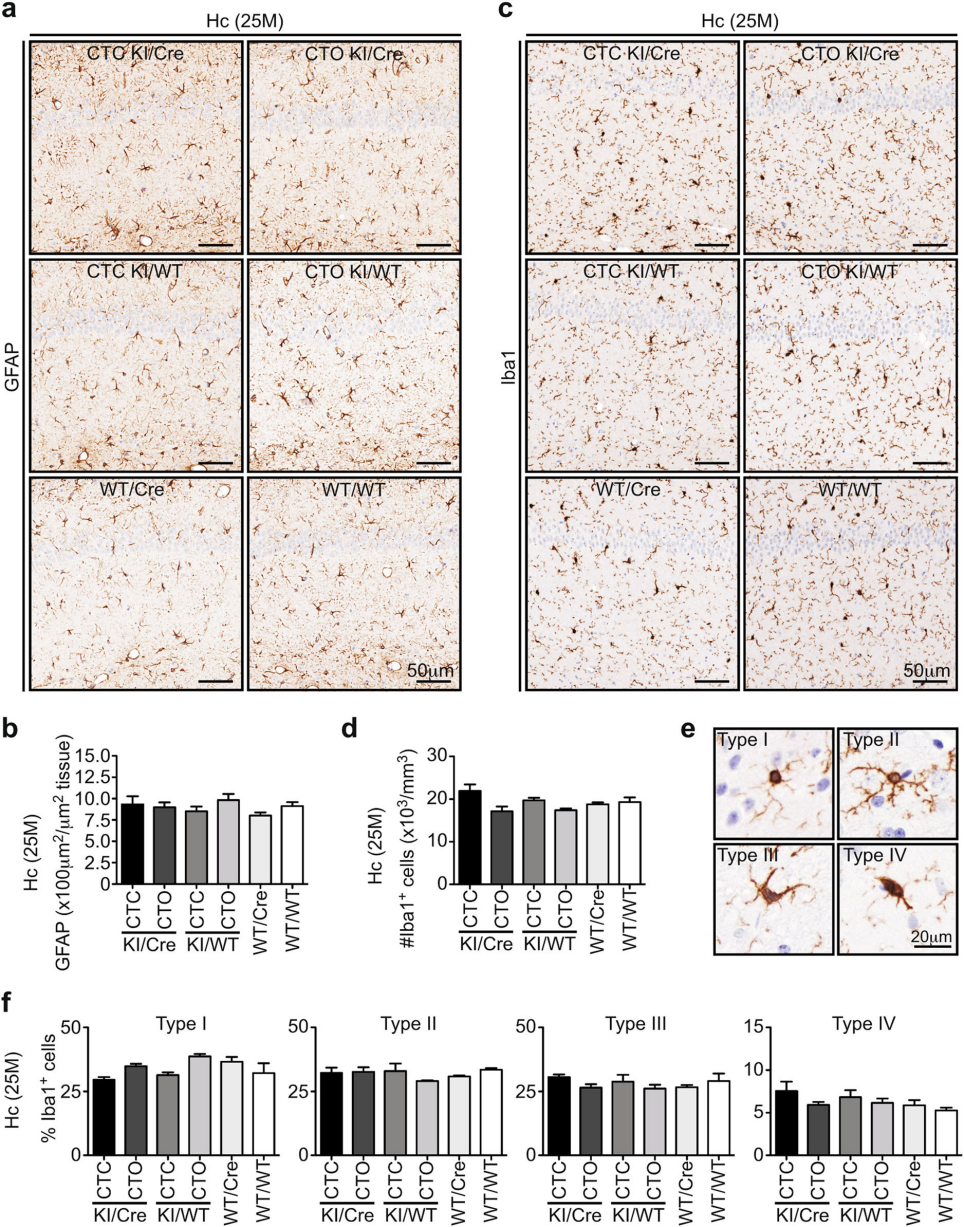
Old CTC KI/Cre mice do not display neuroinflammation in the hippocampus. **a** Representative micrographs and **b** quantifications of 25-month-old CTC and CTO KI/Cre, CTC, and CTO KI/WT, WT/Cre or WT/WT hippocampus tissue sections immunostained with anti-GFAP. **c–f** Representative micrographs and quantifications of 25-month-old CTC KI/Cre, CTO KI/Cre, CTC KI/WT, CTO KI/WT, WT/Cre, or WT/WT hippocampus tissue sections immunostained with anti-Iba1. Data represent the mean ± SEM. *n* = 3/genotype. Statistical evaluations were done with one-way ANOVA followed by a Bonferroni’s post-hoc analysis. Hc hippocampus.

Since previous hCasp6 or TauΔD421 mouse models showed age-related neuroinflammation, 25-month-old brains were immunostained for glial fibrillary acidic protein (GFAP) and ionized calcium binding adaptor molecule 1 (Iba1) (Fig. 5). CA1 astrocytic GFAP^+^ staining area was similar in the six genotypes (Fig. 5a, b), indicating the absence of astrogliosis. Iba1^+^ microglia density in KI/Cre was equivalent to that of controls (Fig. 5c and d). Furthermore, the percentage of types I-IV Iba1^+^ microglia did not differ between genotypes (Fig. 5e, f), indicating the same microglial activation profile. Together, these results indicate that the CTC KI/Cre hippocampus is devoid of neuroinflammation.

### Old CTC KI/Cre mice display slight intraneuronal accumulation of phosphorylated-Tau, and rare hippocampal argentophilic deposits

**Fig. 6:**
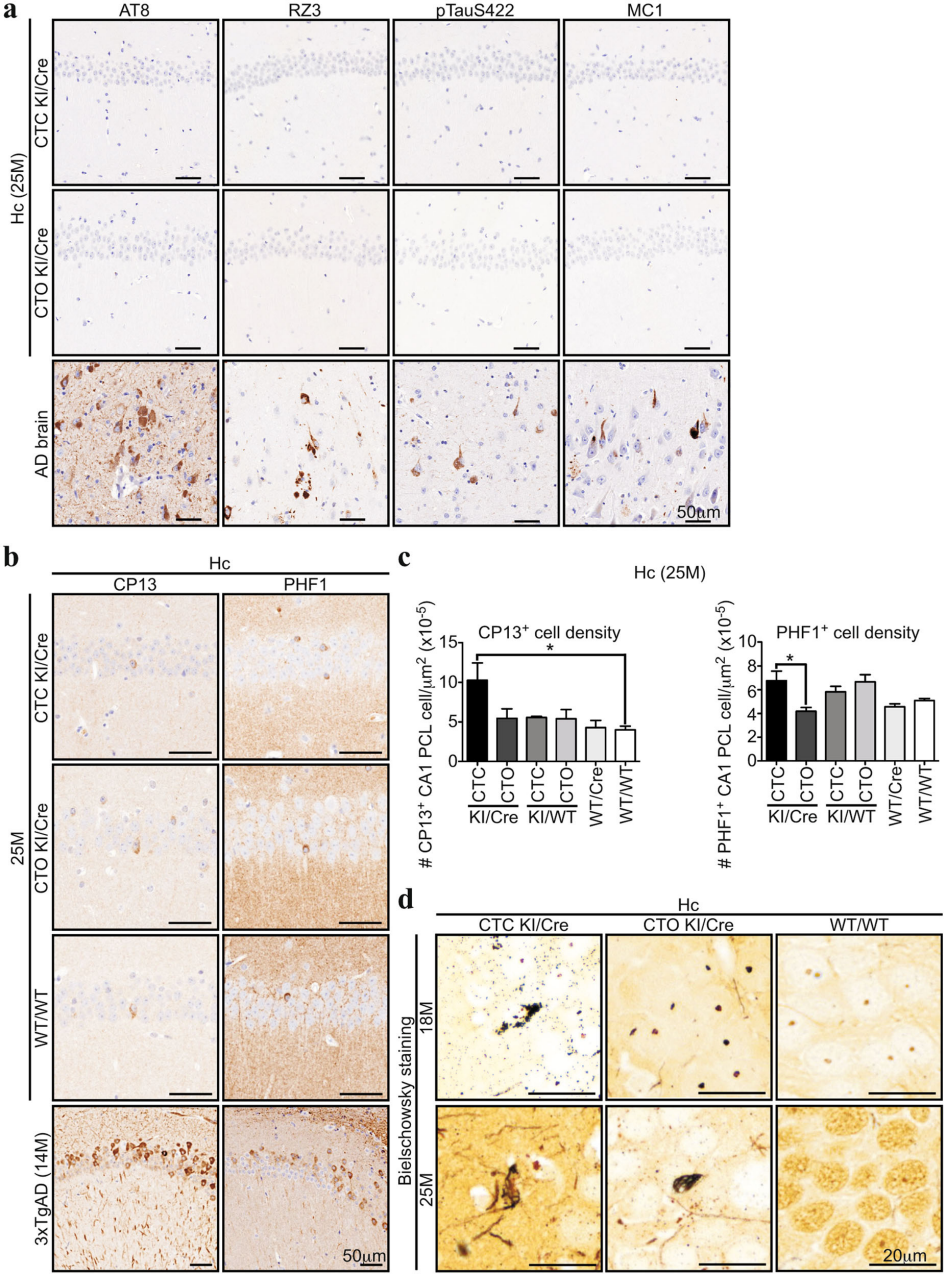
Old CTC KI/Cre mice display a slight intraneuronal accumulation of Tau phosphorylated at S202 and S396/404, and rare argentophilic deposits in the CA1. **a** Representative micrographs of 25-month-old CTC KI/Cre, CTO KI/Cre, or AD brain hippocampus tissue sections immunostained with AT8 (pTauS202/T205), RZ3 (pTauT231), pTauS422, and MC1 (pathologic Tau conformation) antibodies. **b** Representative micrographs of 25-month-old CTC and CTO KI/Cre, WT/WT, or 14-month-old 3xTgAD hippocampus tissue sections immunostained with CP13 (pTauS202) and PHF1 (pTauS396/404) antibodies. **c** Quantifications of the number of CP13 or PHF1 immunopositive cells per μm of in the CA1 PCL of 25-month-old CTC and CTO KI/Cre, CTC, and CTO KI/WT, WT/Cre or WT/WT (*n* = 3/genotype). Data represent the mean ± SEM. Statistical evaluations were done with one-way ANOVA followed by a Bonferroni’s post-hoc analysis. **d** Representative micrographs of Bielschowsky silver staining of 18- and 25-month-old CTC and CTO KI/Cre, or WT/WT hippocampus tissue sections (18 M: *n* = 4/genotype, 25 M: *n* = 3/genotype). Hc hippocampus.

Intracellular Tau accumulation was analyzed in hippocampal sections from 25-month-old mice with antibodies commonly used to detect Tau pathology (Fig. 6 and Supplementary Fig. 7). AT8 anti-pTauS202/T205, RZ3 anti-pTauT231, anti-pTauS422, and MC1 anti-misfolded-Tau immunostainings did not show any immunopositivity in the mouse sections, despite being detected in AD brains (Fig. 6a). In contrast, CP13 anti-pTauS202 and PHF1 anti-pTauS396/S404 immunopositive CA1 pyramidal cell layer (PCL) soma were detected in the brain of all mice genotypes. However, these were not as abundant as CP13^+^ and PHF1^+^ CA1 neurons from 14-month-old 3xTgAD mice (Fig. 6b). The number of CP13^+^ CA1 neurons was increased 2 fold in CTC KI/Cre compared to WT/WT (Fig. 6c). CTC KI/Cre PHF1^+^ CA1 PCL neurons were also increased compared to those in CTO KI/Cre. These differences were not detected by western blot (Supplementary Fig. 7b), suggesting the dilution of the epitope in the extracted proteins. Together, these results indicate that the extent of Tau phosphorylation load in the CTC KI/Cre brains was modest.

Bielschowsky staining was performed to assess the presence of neurodegeneration (Fig. 6d and Supplementary Fig. 7c). Argentophilic fibrillar structures, resembling the stained NFT in AD brains (Supplementary Fig. 7c), were observed in the CA1 PCL of all 25-month-old CTC KI/Cre (*n* = 3) and in one out of three CTO KI/Cre, but not in WT/WT. Only 3–5 argentophilic inclusions were observed per CA1 in CTC and CTO KI/Cre brains. At 18 months, only one CTC KI/Cre displayed silver-stained CA1 inclusions and no staining was observed in 18-month-old or younger CTO KI/Cre and WT/WT (Fig. 6d). The levels of soluble (Supplementary Fig. 8a) and aggregated Tau (Supplementary Fig. 8b) were similar in 25-month-old CTC and CTO KI/Cre, and control hippocampi, as expected due to the rarity of silver-stained structures. Overall, these data suggest that hTau expression induces modest age-dependent neurodegeneration, which is enhanced by Casp6 activity.

### Tau binding to MT is maintained in old CTC KI/Cre hippocampus

**Fig. 7:**
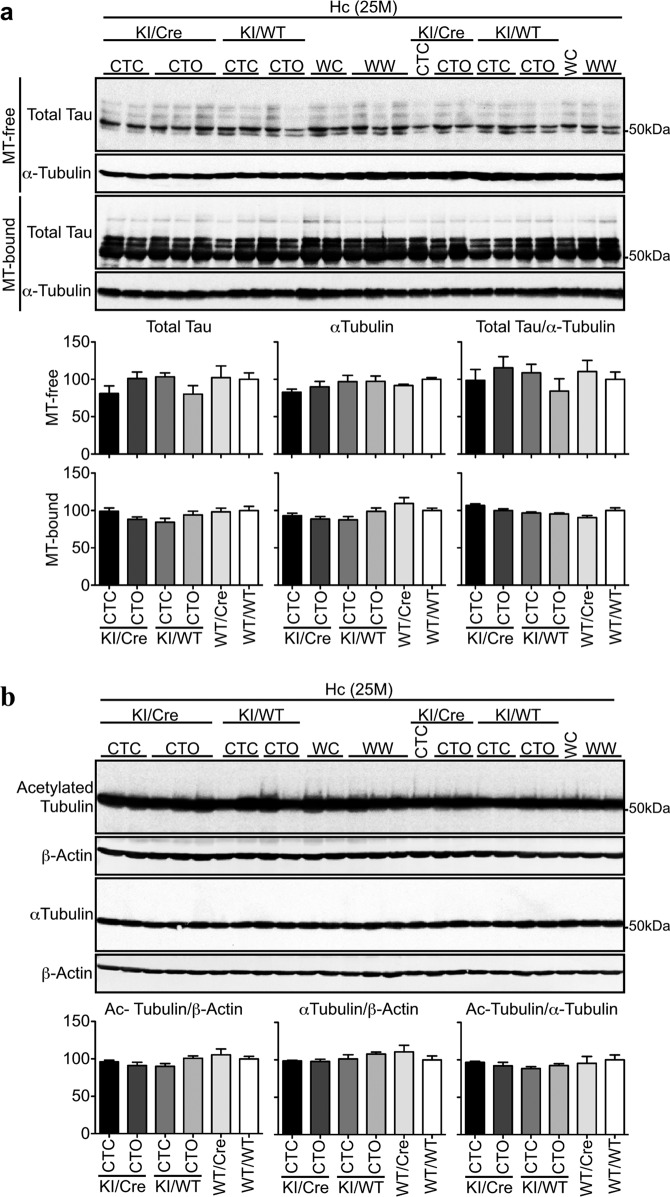
Tau binding to microtubules is maintained in the hippocampus of old CTC KI/Cre mice. **a** Western blot analyses and quantifications of total Tau and α-Tubulin in MT-free and MT-bound protein fractions from the hippocampus (Hc) of 25-month-old CTC KI/Cre (*n* = 3), CTO KI/Cre (*n* = 5), CTC KI/WT (*n* = 4), CTO KI/WT (*n* = 4), WT/Cre (*n* = 3) and WT/WT (*n* = 5). **b** Western blot of 10 μg total proteins from the hippocampus of 25-month-old CTC KI/Cre (*n* = 3), CTO KI/Cre (*n* = 5), CTC KI/WT (*n* = 4), CTO KI/WT (*n* = 4), WT/Cre (*n* = 3), and WT/WT (*n* = 5) with anti-acetylated-Tubulin, anti-α−Tubulin, and anti-β-Actin antibodies. WC: WT/Cre, WW: WT/WT. Data represent the mean ± SEM. One-way ANOVA followed by Bonferroni’s post-hoc.

Hippocampal MT-free and -bound Tau levels were identical in 25-month-old CTC and CTO KI/Cre, and littermates (Fig. 7a). Furthermore, hippocampal Tubulin acetylation, a hallmark of stable MT, was similar in all genotypes (Fig. 7b). Together, these data indicate that the presence of Tau∆D402/D421 in the hippocampus does not alter the ability of Tau to interact with MT.

## Discussion

This work indicates that TauΔD402/D421 are not pivotal for Tau pathogenesis. CTC KI/Cre mice display Tau∆D402/D421 but no significant Tau hyperphosphorylation, misfolding, or aggregation up to 25 months of age. Moreover, CTC KI/Cre brains are devoid of significant neurodegeneration. These results are consistent with the absence of Tau aggregation or cell death in cell lines and drosophila expressing Tau∆D421 (refs. ^45–48^). Mice expressing endogenous Tau non-cleavable at D421 unexpectedly show Tau hyperphosphorylation, memory impairment, and synaptic plasticity deficits, indicating that TauΔD421 could be beneficial in this model or that the D421N mutation altered Tau function^32^. However, our work contradicts studies implicating Tau∆D402/D421 in Tau aggregation and toxicity in AD brains^7–13^, transgenic animal models^24–29,31,49^, neuronal cells^17–22^, and recombinant proteins^7,23^. Specifically, transgenic or AAV-directed Tau∆D421 expression in mice induces Tau pathology, neuroinflammation and cognitive deficits^28,29,31^.

CTC mice are particularly relevant for AD research. Tau 0N4R is expressed 3–4 weeks after birth when Tau expression naturally shifts from 3 to 4 R isoforms^50^. Tau 0N4R overexpression mimics the increase of this isoform in AD^51,52^. The CTC model co-expresses hCasp6 in tandem with hTau to assure proteins co-localization in the same cell, as observed in AD neurons^8^. Furthermore, hTau and active Casp6 expression occurs in a cell-type- and regional distribution-specific manner as observed in AD brains^11^. Lastly, the long-term survival of CTC mice allows studies in aging animals. The difference between CTC and hTau∆D421 expressing mice could additionally be due to the absence of Tau N-terminus in TAU62 mice^29^, massive Tau∆D421 levels^28,29,31^, or precocious Tau 0N4R expression^28,50,53^. Murine Tau unlikely affects Tau∆D402/D421 in CTC mice since TAU62, TauC3, and AAV-TauΔD421 infected mice also express endogenous Tau^28,29,31^. Furthermore, the mouse background can be excluded since CTC, AAV-Tau∆D421 mice, and TAU62 models are all on the C57BL/6 genetic background.

Nevertheless, our results do not exclude that additional stressors could implicate Casp6-cleaved Tau in AD pathogenesis. Tau fragments cleaved at D25, K44, R230, N255, D314, and N368 that have been identified in AD brains remain to be investigated^6,15,16^. In addition, other post-translational modifications, especially Tau hyperphosphorylation, might be required to convert Casp6-cleaved Tau into a pathological entity.

The lack of phenotype in CTC mice was surprising since the ACL mouse, expressing the same amount of Casp6, develops age-dependent neurodegeneration, neuroinflammation, and cognitive deficits that are reversed by Casp6 inhibitors^12,36,54^. Consistent with our results, Tau overexpression prevents cell death and decreases Casp6 activity in serum-deprived neurons^55^. Moreover, Tau∆D421 binds MT more efficiently than full-length Tau^47^, suggesting that Tau∆D421 may prevent neuronal cytoskeleton destabilisation. Together, these results suggest that Tau overexpression in CTC may have directly buffered Casp6 overactivity by limiting Casp6 accessibility to other substrates or activating protective signalling pathways against Casp6 toxicity. Alternatively, TauΔD402/D421 may be beneficial under certain conditions. However, Casp6 was not completely inhibited by Tau since Tub∆Casp6 was observed in CTC mice. Comparing CTC and ACL brain proteomes may help identify substrates critical for Casp6-mediated toxicity and design new Casp6 inhibitors.

The absence of Tau pathology, neurodegeneration, neuroinflammation, and cognitive deficits in CTO mice was also unexpected since these features are commonly observed in transgenic models overexpressing non-mutated full-length hTau^56–62^. Random genomic insertions, expression of some Tau isoforms at the wrong developmental stage and at extra-physiological levels, could explain Tau toxicity in some mice. Our models avoid these potential problems by conditionally expressing Tau 0N4R, the predominant Tau species in adult murine axons^63^, from a knocked-in transgene in the hypoxanthine-guanine phosphoribosyl transferase *(Hprt)* locus. Our results are consistent with data from transgenic mice in which the entire genomic sequence of hTau has been inserted to allow normal physiological expression and alternative splicing. These mice are exempt from Tau pathology, neurodegeneration and memory deficits^64,65^.

The CTC and CTO models could be useful to assess region- and time-specific hTau expression in brains simply by using another Cre mouse. These may also be useful to study Tau propagation. Furthermore, hCasp6 cDNA in the CTC could be replaced by other genes to assess their implication in Tau function, structure and pathology.

In conclusion, this study suggests that in vivo Tau cleavage by Casp6 in CA1 and cortical neurons is insufficient to induce Tau pathogenesis and might not be an appropriate AD therapeutic target. Since Casp6 has many neuronal protein substrates, it is reasonable to conclude that Casp6-mediated damage occurs in many pathways that contribute to neurodegeneration and it may be more important to target Casp6 rather than its substrates in AD.

## Materials and methods

### Mice

All animal procedures followed the Canadian Council on Animal Care guidelines and were approved by the animal care committees of McGill University (protocols #2009-5727, #2011-6027, and #2016-779) and Université de Montréal (protocol #19-045). Experimental mice were generated and aged at the Institute for Research in Immunology and Cancer (IRIC) of the Université de Montréal pathogen-free animal facility, and transferred to the Lady Davis Institute (LDI) animal facility for behavioural experiments two weeks before experiments for adjustment to a reverse light cycle. Animals were group-housed (2-3 animals per cage) in standard macrolon cages (40 × 25 × 20 cm) with contact bedding (7907, Envigo Teklad Lachine, QC, Canada), one Nestlet^®^ (NES3600, Ancare Corporation, Bellmore, NY, USA) and one cardboard house (XKA2455, Ketchum manufacturing, Brockville, ON, Canada) in a 50–70% humidity and 20–24 °C temperature-controlled room. Sterile food (2920X, Envigo Teklad) and water were available ad libitum.

#### Generation of a mouse model conditionally expressing hTau in tandem with hCasp6 in cortical and hippocampal CA1 pyramidal neurons: the CTC model

CaMKIIα-Cre-dependent hTau and hCasp6 expressing mouse, or CTC, was created (genOway, Lyon, France) to express the hTau 0N4R isoform with a self-activated form of hCasp6 under Cre/loxP recombination in the cortical and hippocampal CA1 pyramidal neurons. The transgene was composed of the ubiquitous cytomegalovirus immediate early enhancer fused to the chicken β-Actin promoter, a floxed STOP cassette, and hCasp6 cDNA followed by hTau cDNA (Supplementary Fig. 1a). An internal ribosomal entry site sequence was inserted between hCasp6 and hTau cDNA to allow the translation of both proteins from a bicistronic mRNA. The hCasp6 cDNA lacked its pro-domain to promote self-activation upon expression in mammalian cells^66^, was his-tagged and was flanked with Flippase recognition target sites to allow flippase (Flp)-dependent conditional deletion. The transgene was inserted into the Quick knock-In vector from genOway, and then introduced into the *Hprt* locus of 129Ola (E14) embryonic stem (ES) cells. E14 ES cells display a deletion of 35 kb upstream of the *Hprt* gene intron 2, which renders these cells unable to grow in culture medium containing hypoxanthine, aminopterin and thymidine. Since the targeting vector contained the wild-type *Hprt* sequence, targeted insertion of the vector repaired the *Hprt* gene deletion. Consequently, correctly recombined E14 ES cells were selected using medium containing hypoxanthine, aminopterin, and thymidine, and correct homologous recombination was validated by PCR and southern blot. Targeted insertion into the *Hprt* locus was chosen to allow insertion of a single transgene copy, to avoid potential defects due to random genomic insertions and to protect the transgene from gene silencing. After transfection and selection, E14 ES cells were injected into C57BL/6 J blastocysts. The transgenic animals were then crossed with CaMKIIα-regulated Cre T29.1 heterozygous mice (Jackson Laboratories, Bar Harbour, ME, USA) to generate mice expressing hTau and hCasp6 exclusively in the CA1 pyramidal neurons of the hippocampus and scattered forebrain neurons 2-3 weeks after birth, according to Cre expression pattern of the T29.1 model^67^. Mice were produced by in vitro fertilization to avoid eventual germ line transmission of the excised-STOP-cassette transgene related to Cre expression in the testis of T29.1 mice^36,68^. Since the hCasp6 and hTau cDNA were inserted into the *Hprt* locus of the X chromosome, only males were used to avoid lionization effects. Human Casp6-hTau expressing progeny, carrying both the CaMKIIα-Cre and the hCasp6-hTau transgenes, were called CTC KI/Cre mice. Control littermates were identified as: CTC KI/WT: animals carrying the hCasp6-hTau cDNA without the CaMKIIα-Cre transgene, WT/Cre: heterozygous CaMKIIα-Cre mice devoid of the hCasp6 and hTau cDNA, and WT/WT: wild-type animals.

#### Generation of a mouse model conditionally expressing hTau only in the cortex and CA1 region of the hippocampus: the CTO model

CaMKIIα-Cre-dependent hTau only expressing mouse, or CTO, was created to express hTau 0N4R under Cre/loxP recombination in the cortex and hippocampal CA1 pyramidal neurons. Mice carrying the hCasp6-hTau cDNA knocked-in in the *Hprt* locus (described above) were crossed with C57BL/6 J Flp-deleter mice to specifically remove the hCasp6 cDNA sequence. The offsprings were screened by PCR and southern blot for the presence of the Flp transgene and analysis of the *Hprt* locus. Animals devoid of Flp and carrying the Flp-excised recombined *Hprt* locus were then crossed with CaMKIIα-Cre T29.1 heterozygous mice. Male progeny was identified as: CTO KI/Cre: hTau expressing mice carrying both the CaMKIIα-Cre and the hTau transgenes, CTO KI/WT: animals carrying the hTau cDNA without the CaMKIIα-Cre transgene, WT/Cre: heterozygous CaMKIIα-Cre mice, and WT/WT: wild-type animals.

Animals were produced by in vitro fertilizations over a period of one month, and randomly assigned for behavioural testing at 3, 9, 18, and 25 months of age. Mice were then sacrificed and randomly assigned to either histological or biochemical analysis, independent of behavioural performance. Two-month-old animals were also sacrificed for molecular biology and biochemistry analyses. Sample sizes were based on previous studies showing cognitive deficits in the ACL mice expressing equivalent levels of hCasp6 only.

#### Other mouse models

The ACL mouse model expressing hCasp6 under CaMKIIα-Cre-dependent expression was previously generated to express a self-activated form of hCasp6 under Cre/loxP recombination in the cortex and hippocampal CA1 pyramidal neurons^12,36^. Briefly, a transgene composed of the ubiquitous cytomegalovirus immediate early enhancer fused to the chicken β-Actin promoter, hCasp6 cDNA and an upstream loxP-flanked STOP sequence was inserted into the *Hprt* locus with the Quick Knock-In technology from genOway. The resulting transgenic mice were then crossed with T29.1 CaMKIIα-Cre animals. ACL mice were on a C57BL/6 J background. ACL KI/Cre mice showed age-dependent episodic and spatial memory impairments, neurodegeneration, and neuroinflammation.

Wild-type C57BL/6 J azoxymethane/dextran sulphate sodium (AOM/DSS) treated animals were generated previously^69^.

Casp6 KO #006236 and Tau KO #004779 mice on a C57BL/6 J background were obtained from Jackson Laboratories.

Tissues from the 3xTgAD mice were received from Dr Emmanuel Planel (U. Laval, QC, Canada) who obtained the mice from Dr Frank LaFerla (University of California, Irvine, USA) and are on a C57BL/6;129 × 1/SvJ;129S1/Sv background. These transgenic mice harbour knocked-in human presenilin-1 with the M146V mutation, and the human Amyloid Precursor Protein (APP695 isoform) cDNA with the Swedish double mutation (KM670/671NL) and the hTau 0N4R with the P301L mutation cDNA, both under the control of the Thy1.2 promoter.

### Genotyping of experimental mice

Tail biopsies were incubated in 300 μL of 50 mM NaOH for 20 min at 99 °C, then 25 μL of 1 mM Tris pH: 8.0 were added to the samples. Tail debris were pelleted by 10 min centrifugation at 15900 × *g* and the supernatant was used for PCR with GoTaq^®^ DNA polymerase according to the manufacturer’s protocol (New England Biolabs, Whitby, ON, Canada). The primers 5’-ATCCGAAAAGAAAACGTTGA-3’ and 5’-ATCCAGGTTACGGATATAGT-3’ were used to amplify a 650 bp sequence from the CamKIIα-Cre transgene. To screen samples for the presence of the hCasp6-hTau or hTau cDNA, the primers were: forward for wild-type *Hprt*: 5’-CCTCCAGAAGCTCCAATGTGGATGC-3’, forward for recombined *Hprt*: 5’-CGGTGGGACATTTGAGTTGCTTGC-3’, and reverse: 5’-ACGTCAGTAGTCATAGGAACTGCGGT CG-3’. This PCR generated a 1 219 bp product corresponding to the wild-type *Hprt* allele, and a 340 bp product corresponding to the recombined *Hprt* alleles. To discriminate between the CTC and CTO models, the primers were: forward: 5’-CATGGTAAGTAAGCTTGGGCTGCAGG-3’, reverse for recombined *Hprt* from CTC*:* 5’-CATCTGCGTGGCTAACAGTTGACACC-3’, and reverse for recombined *Hprt* from CTO: 5’-GCCAAAAGACGGCAATATGGTGG-3’. This PCR generated a 488 bp amplicon corresponding to the recombined *Hprt* from CTC and a 228 bp amplicon corresponding to the recombined *Hprt* from CTO.

### Behavioural tests

Two days after transfer from the IRIC to the LDI, the animals were handled 5 min daily for 12 days to reduce fear and anxiety due to the presence of the experimenter. Groups of mice were tested at 3 (CTC KI/Cre *n* = 15, CTO KI/Cre *n* = 15, CTC KI/WT *n* = 15, CTO KI/WT *n* = 16, WT/Cre *n* = 16 and WT/WT *n* = 15), 9 (CTC KI/Cre *n* = 16, CTO KI/Cre *n* = 15, CTC KI/WT *n* = 13, CTO KI/WT *n* = 15, WT/Cre *n* = 15 and WT/WT *n* = 16), 18 (CTC KI/Cre *n* = 13, CTO KI/Cre *n* = 14, CTC KI/WT n = 15, CTO KI/WT *n* = 17, WT/Cre *n* = 14 and WT/WT *n* = 17), and 25 months of age (CTC KI/Cre *n* = 9, CTO KI/Cre *n* = 17, CTC KI/WT *n* = 14, CTO KI/WT *n* = 16, WT/Cre *n* = 10, and WT/WT *n* = 18). They were subjected to the openfield test on day 1, and to the novel object recognition test, 24 h later. Barnes maze testing was performed on day 5 to day 10. The animals were sacrificed on day 14. The experimenter was blinded to the genotype. All animals were re-genotyped after death.

#### Openfield

Mice locomotor activity was video recorded during a 5 min exploration session in an empty box (#60111, Stoelting Co, Wood Dale, IL, USA). The HVS 2100 automated video tracking system (HSV Image, Hampton, UK) divided the field in 16 virtual zones (4 rows × 4 columns) and analyzed the distance travelled, the percentage of time moving, the percentage time spent in the periphery, the percentage of virtual zones used and the number of entries into each zone. One 3-month-old CTO KI/Cre mouse was excluded because it moved less than 20% of the time.

#### Novel object recognition

Mice were placed into the openfield box and were pre-exposed during 5 min to two identical objects (62020, Stoelting Co) located in the middle of the northwest and southeast quadrants of the box, equidistantly from the box corners and from each other. Two hours later, the animals were presented with the familiar object and a novel object for 5 min. The position of the novel object was interchanged between the animals to avoid any bias related to a preference in the location of the new object and the use of potential spatial cues. The number of times touching each object was manually recorded and the mice performances were expressed as the discrimination index, which represents the number of time touching the novel object minus the touches to the familiar object divided by the total number of touches. A mouse was considered as impaired when the percentage of touches to the novel object was ≤55%, which represents a discrimination index ≤0.1. The comparison of the number of impaired mice between two genotypes of the same age was done with the two-tailed Fisher’s exact test. One 3-month-old CTO KI/Cre mouse that did not touch either object was excluded.

#### Barnes maze

Spatial memory of the ACL mouse model was initially characterized with the Morris water maze^12^. However, Barnes maze testing was used in this study to avoid artificial increase in Tau phosphorylation levels related to water immersion-induced hypothermia. This protocol has previously been successfully used to assess spatial memory in rodents, including in Casp6 overexpressing mice^36,37^. The platform consisted in a 91 cm diameter circular dry-land maze containing 20 holes of 5 cm in diameter and elevated to a height of 90 cm from the ground. Spatial cues consisting in geometrical black pictures were placed on the white walls surrounding the maze. Mice were trained to escape from an elevated circular platform to a small static dark chamber located under one of the 20 holes around the platform perimeter (target hole). For each trial, the mice were placed in the middle of the platform under a dark box, and 10 s after the box was removed and the mice were exposed to bright light provided by a 150-watt incandescent light bulb, and a buzzer was turned on. Once in the escape chamber, the buzzer was turned off and the target hole covered. Barnes maze was conducted in three phases: adaptation (day 0), spatial acquisition (days 1–4) and test (day 5). The locations of the spatial cues and escape chamber were kept constant throughout the study. On day zero, the mice were free to explore the maze for 60 s and to stay in the escape chamber for 120 s. On days 1–4, the mice were subjected to 4 daily trials of 180 s each with an inter-trial interval of 15 min. If the mouse did not successfully escape the maze within 180 s, it was gently led into the escape chamber and left inside for 60 s. The latency to reach the target hole (primary latency), the number of errors to the first encounter of the escape hole (primary errors), the number of total errors and the latency (time spent before the mouse enters the escape hole) were recorded. On day 5, the mice were submitted to a single probe test. The target hole was blocked and the mice were allowed to explore the maze for 90 s under the same aversive conditions. The primary latency, the number of primary errors and the number of nose pokes for each hole, reflecting the hole preference, were recorded. Mice were tracked with the HVS 2100 automated video software throughout the testing. A mouse was considered as impaired if the percentage of touches to the target hole during the probe test was less than the touches to any other hole. The comparison of the number of impaired mice between two genotypes of the same age was done with the two-tailed Fisher’s exact test. Two 25-month-old CTC KI/Cre, one 3-month-old WT/WT, and one 25-month-old WT/WT were excluded because they froze during the training sessions and/or the probe test.

### Total RNA extraction and Reverse-Transcriptase Polymerase Chain Reaction

RNA was isolated with Qiazol and purified using the miRNeasy mini kit (Qiagen, Valencia, CA, USA). Total RNA was treated with deoxyribonuclease RNA-qualified (RNAse RQ1, Promega, Madison, WI, USA) prior to cDNA synthesis. Reverse transcription (RT) was performed with the First Strand cDNA Synthesis Kit (Qiagen) following the manufacturer’s protocol. Quantitative real-time polymerase chain reaction (qPCR) experiments were performed with SYBR Green Taq Mastermix (Quanta BioSciences, Gaithersburg, MD, USA) in a real-time cycler ABI 7500 Fast Block (Applied Biosystems, Foster City, CA, USA). The primers were 5’-CGATGTGCCAGTCATTCCTT-3′ and 5’-TCTAAGGAGGAGCCATATTTT C-3′ for hCasp6; 5’-TGATGGAAGATCACGCTGGG-3’ and 5’-CCCTCTTGGTCTTGGTGCAT-3’ for hTau; 5’-TCTCAGGTACTGACGGTGGACCAGCTTGCATGATCTCC-3′ and 5′-GTGAAA CAGCATTGCTGTCACTT-3’ for Cre; 5’-GTAACCCGTTGAACCCCAT-3’ and 5’-CCATCCAATCCGTA GTAGCG-3’ for 18 S. Results were analyzed according to the 2^−ΔCT^ method^70^. Quantification was blinded to genotype.

### Multiplex PCR analysis of the STOP-cassette loci of the transgenic mice

Multiplex PCR were performed on 30 ng genomic DNA extracted with Trizol (Invitrogen, Carlsbad, CA, USA) according to the manufacturer’s protocol. Primers were designed by genOway. The experiment was performed four times.

#### CTC model

5’-ACCATGTTCATGCCTTCTTCTTTTTCC-3’, 5’-TCAGGAAGACACACACAAAGCAAT CG-3’, and 5’-AAACCAAGAAGTGCCAGAAATAACAGTAGC-3’ primers were used to amplify a 450 bp sequence of the non Cre-excised recombined *Hprt* allele (allele with the STOP-cassette sequence) and a 627 bp sequence of the Cre-excised recombined *Hprt* allele (allele without the STOP cassette). Amplifications were conducted with the 2x Phire tissue direct PCR master mix (ThermoFisher Scientific, Mississauga, ON, Canada) for 35 cycles of 95 °C for 5 s, 64 °C for 5 s, and 72 °C for 30 s, with a final 72 °C extension phase of 60 s.

#### CTO model

5’-CTAGAGCCTCTGCTAACCATGTTCATGC-3’, 5’-AAACATGTCCCAGCTCCAAGAA ACG-3’, and 5’- GCCAAAAGACGGCAATATGGTGG-3’ primers were used to amplify a 276 bp sequence of the non Cre-excised recombined *Hprt* allele and a 334 bp sequence of the Cre-excised recombined *Hprt* allele. Amplifications were conducted with the M300 GoTaq DNA polymerase and the corresponding 5x green buffer from Promega for 35 cycles of 95 °C for 30 s, 64 °C for 30 s, and 72 °C for 30 s, with a final 72 °C extension phase of 5 min.

### Protein extraction

Mice were sacrificed by cervical dislocation without anaesthesia to avoid any transient changes in Tau phosphorylation^71^. Peripheral tissues and brains were quickly dissected on ice and frozen on dry ice. Frozen samples were directly homogenized in modified cold radioimmunoprecipitation assay (RIPA) buffer (50 mM Tris-HCl pH: 7.4, 1 mM ethylenediamine-tetraacetic acid (EDTA), 150 mM NaCl, 1% NP-40, 0.25% Na-deoxycholate, 1 mM sodium orthovanadate, 1 mM sodium fluoride (NaF), 1 mM phenylmethylsulfonyl fluoride (PMSF), 10 μL/mL P8340 protease inhibitors cocktail (Sigma–Aldrich, Oakville, ON, Canada)) with a mechanical homogenizer (Omni International, Kennesaw, GA, USA), and centrifuged at 18440 × *g* for 20 min at 4 °C. The resulting supernatants corresponding to the total RIPA proteins were kept at −80 °C for analyzes.

Aggregated Tau was isolated as described^72^. Total RIPA proteins were adjusted to 1% N-lauroylsarcosine (sarkosyl), incubated for 30 min at room temperature with constant orbital shaking, and centrifuged at 100 000 g for 60 min at 20 °C. The supernatants were discarded and the sarkosyl-insoluble pellets re-suspended in sample buffer (NuPAGE LDS (Invitrogen) containing 5% of 2-β-mercaptoethanol, 1 mM sodium orthovanadate, 1 mM NaF, 1 mM PMSF, 10 μL/mL P8340 Sigma protease inhibitors cocktail), boiled for 5 min and kept at −20 °C until western blot analyzes. Protein extractions were done blinded to genotype.

### Endogenous microtubule-binding assay

The association of endogenous Tau to endogenous MT was analyzed according to the endogenous MT binding assay^73^. Mice were killed by cervical dislocation without anaesthesia and the brains dissected out. Fresh cortices and hippocampi were immediately homogenized together in 37 °C extraction buffer (80 mM MES pH: 6.8, 1 mM MgCl_2_, 2 mM ethylenediamine-tetraacetic acid (EGTA), 30% glycerol, 0.1% Triton X-100, 1 mM PMSF, 1 mM sodium orthovanadate, 1 mM NaF, 10 μL/mL P8340 Sigma Protease Inhibitor Cocktail) with a pre-warmed (37 °C) mechanical homogenizer. The samples were immediately centrifuged at 3000 × *g* for 2 min at 25 °C, and an aliquot of the supernatant, identified as the total fraction, was centrifuged at 100000 × *g* for 20 min at 25 °C. The supernatant corresponding to the MT-free protein fraction was removed, and the remaining pellet corresponding to the MT-bound protein fraction was re-suspended in extraction buffer. Total, MT-free and MT-bound protein fractions were stored at −80 °C until processing.

### Immunoprecipitation

Frozen tissues were homogenized with a mechanical homogenizer in sterile immunoprecipitation (IP) buffer (25 mM Tris-HCl pH: 7.4, 150 mM NaCl, 1 mM EDTA, 1% NP-40, 5% glycerol, 38 μg/mL 4-(2-aminoethyl)-benzenesulfonyl fluoride hydrochloride (AEBSF), 0.5 μg/mL leupeptin, 0.1 μg/mL tosyl-L-lysyl-chloromethane hydrochloride (TLCK), 0.1 μg/mL pepstatin, 1 mM sodium orthovanadate, 1 mM NaF, 1 mM PMSF), and centrifuged at 4 °C for 20 min at 18440 × *g*. Five hundred μg of protein were pre-cleaned by orbital incubation with 50 μL protein A Sepharose beads (ThermoFisher Scientific) for 2 h at 4 °C. After centrifugation at 1000 × *g* for 1 min at 4 °C, pre-cleaned lysates were incubated for 4 h at 4 °C with 1/100 10635 anti-TauΔD402 antibody^8^ or equivalent volume of IP buffer, and then protein A Sepharose beads were added and the incubation was continued overnight. The immune complexes were recovered by centrifugation and rinsed five times with cold sterile phosphate-buffered saline (PBS) containing 1 mM PMSF. This experiment was replicated twice.

### In vitro digestion by recombinant active Casp6

Human cerebellum (obtained with written informed consent from the University of Toronto Institutional Review Board by Dr C. Bergeron)^8^, or murine Casp6 KO brainstem RIPA proteins were boiled for 5 min at 95 °C and then centrifuged for 20 min at 18440 × *g* at 4 °C. This method is used to isolate proteins resistant to heat, including Tau. Thus, other endogenous proteins, like caspases, were precipitated during the boiling process and eliminated from the supernatant. The pellets were re-suspended in sample buffer, boiled for 5 min and stored at −20 °C. The heat-resistant proteins in the supernatant were precipitated with acetone (1:4 ratio, −20 °C overnight)^74^, re-suspended in Stennicke’s buffer (SB) (20 mM piperazine-N, N-bis (2-ethanesulfonic acid) (PIPES) pH: 7.2, 30 mM NaCl, 1 mM EDTA, 0.1% 3-[(3-cholamidopropyl) dimethylammonio]-1-propanesulfonate (CHAPS), 10% sucrose) and submitted to in vitro digestion by recombinant hCasp6 (generated by Dr Agne Tubeleviciute-Aydn in our lab) for 1 or 4 h at 37 °C. The reaction mix consisted of 330 ng active site titrated recombinant Casp6p20p10 prepared from the pET23b cDNA construct^43^, 100 μg brain heat-resistant proteins extract or 189 ng rTau 0N4R (Biolegend, San Diego, CA, USA), SB and freshly added 10 μM dithiothreitol. The digestion was replicated four times for rTau and three times for heat-resistant extracts.

### Western blot

Samples were prepared in sample buffer. Proteins were separated on sodium dodecyl sulfate-polyacrylamide gel electrophoresis (SDS-PAGE) gels, blotted onto nitrocellulose or polyvinyl fluoride membranes, and blocked 1 h at room temperature with 5% non-fat dry milk in PBS containing 0.1% Tween 20 (PBS-T). Membranes were then washed three times 10 min with PBS-T, and probed overnight at 4 °C with primary antibodies diluted in SuperBlock^®^ blocking buffer (ThermoFisher Scientific) (Table 1). After three washes of 10 min with PBS-T, the membranes were incubated for 1 h at room temperature with the corresponding horseradish peroxidase (HRP)-linked secondary antibody diluted in PBS-T containing 5% non-fat dry milk. For monoclonal anti-Tau antibodies, an HRP-coupled antibody directed against the light chain of murine immunoglobulins was used as secondary antibody (#AP200P, Millipore). Membranes were revealed by enhanced chemiluminescence (GE Healthcare Life Sciences, Mississauga, ON, Canada) in a Fujifilm LAS4000 imaging system (Fujifilm USA, Valhalla, NY, USA). Densitometric analyses were performed with Image Gauge analysis software 3.0. (Fujifilm USA).

**Table 1:** Antibodies used in this study.

Antibody	Epitope	Supplier	Catalog #
β-Actin		Sigma	A5441
Caspase-6	Caspase-6 small subunit	Cell signaling	9762
human Caspase-6	Caspase-6 160-210	Lifespan Bioscience	LS-B477
Active Caspase-6	Caspase-6∆D179	Biovision	3156
6xhis-tag	6xhis	My Biosource	MBS194394
Active Caspase-3	Caspase-3∆N175	Cell signaling	9661
AT8	pTauS202/T205	Thermo Fisher Scientific	MN1020B
CP13	pTauS202	Dr P. Davies	
DA9	Tau 102-140	Dr P. Davies	
PHF1	pTauS396/404	Dr P. Davies	
RZ3	pTauT231	Dr P. Davies	
pTauS422	pTauS422	Abcam	ab192205
MC1	Tau 5-15/312-322	Dr P. Davies	
Tau12	Tau 9-18 (human)	Millipore	MAB2241
Tau-C6g	Tau∆D13	Dr N. Kanaan	
10635	Tau∆D402	Dr A.C. LeBlanc	
TauC3	Tau∆D421	Millipore	36-017
GFAP		Dako	Z0334
Iba1		Wako	019-19741
Tub∆Casp6	Tub∆D438	Dr A.C. LeBlanc	
Acetylated Tubulin		Sigma–Aldrich	T7451
αTubulin		Cell Signaling	3873
anti-mouse HRP		Jackson Immunoresearch Laboratories	115-035-164
anti-rabbit HRP		Dako	P0217
anti-mouse LC Ig HRP		Millipore	AP200P

### Immunohistochemistry

Mice were anesthetised with isoflurane, perfused with cold 0.9% saline and 4% paraformaldehyde in 0.2 M PBS using a peristaltic pump (Thermo Fisher Scientific). The dissected brains were post-fixed for 24 h in 10% neutral-buffered formalin (Thermo Fisher Scientific), embedded in paraffin blocks, and 4 μm sections cut at the IRIC histology core facility (Université de Montréal, Montréal, CA), with three consecutive sections per slide. Brain sections were deparaffinized, hydrated, and incubated for 20 min at 97 °C in the Pascal Cytomation apparatus (Dako, Burlington, ON Canada) with Tris-EDTA antigen retrieval buffer (10 mM Tris Base, 1 mM EDTA, 0.05% Tween-20, pH: 9.0). Immunostainings were performed with the Autostainer Plus automated slide processor and the EnVision Flex system (Dako) as previously described^11^ with the following antibodies (Table 1): 1/8 000 anti-GFAP, 1/2 000 anti-Iba1, 1/5 000 anti-hCasp6, 1/5 000 anti-Tub∆Casp6, 1/100 Tau-C6g (anti-Tau∆D13)^75^, 1/25 000 anti-Tau∆D402, 1/1 000 anti-Tau∆D421, 1/100 anti-cleaved-Casp3 (∆Casp3), 1/100 anti-cleaved Casp6 (∆Casp6), 1/500 anti-6xhis-tag, 1/1 000 anti-pTauS202/T205, 1/1 000 anti-pTauS422, 1/1 000 anti-pTauT231, 1/10 000 anti-pTauS396/S404, 1/1 000 MC1 anti-Tau, 1/10 000 anti-pTauS202. Slides were counterstained with hematoxylin, dehydrated, coverslipped with Permount mounting medium (Thermo Fisher Scientific) and digitally scanned with the MIRAX SCAN scanner (Zeiss, Don Mills, ON, CA). Sections from AOM/DSS-treated wild-type mice and human fetal stomach (BioChain, Institute Inc., Newark, CA, USA) were used as positive controls for ∆Casp3, and sections from human AD brain (kind gift from Dr D.A. Bennett, Rush Medical School, Chicago, Il, USA; grants P30AG10161 and R01AG15819), Casp6 KO, Tau KO, and 3xTgAD mice were used as controls for hCasp6 and/or Tau immunostainings.

Astrocytic GFAP and microglial Iba1 immunopositivity were quantitated on three brain sections per slide and on three slides spaced 56 μm apart. The area of GFAP staining was quantified with Image J software (NIH, Bethesda, MD, USA) and expressed as μm^2^ staining/μm^2^ of tissue. The number of total Iba1^+^ cells/mm^3^ tissue was quantified with Image J software, and Iba1^+^ cells were submitted to morphological characterization according to scoring schemes^76^. Quantifications were blinded to genotype.

### Bielschowsky silver staining

Bielschowsky silver staining was performed with the ab245877 Bielschowsky Silver Stain Kit from Abcam (Cambridge, UK) according to the manufacturer’s protocol. Briefly, formalin-post-fixed, paraffin-embedded 4-μm thick brain sections were deparaffinized, hydrated, incubated in a warm 20% silver nitrate solution for 15 min at 40 °C, and rinsed three times 3 min in water. The slides were then incubated for 30 min in a warm (40 °C) ammonia hydroxide-cleared silver nitrate solution before incubation for 1–2 min in freshly-made developer solution (8 drops of 20% formalin, 8 drops of citric acid, 4 drops of nitric acid, in 50 mL H_2_O). The slides were then immediately placed in 0.2% ammonium hydroxide for 2 min. The sections were rinsed three times 2 min in water and incubated for 2 min in 5% thiosulfate solution. The slides were then dehydrated, coverslipped with Permount mounting medium (Thermo Fisher Scientific) and digitally scanned with the MIRAX SCAN scanner (Zeiss). Brain sections from human AD were used as positive control (Dr D.A. Bennett). Staining and analyses were blinded to genotype.

### Golgi-Cox staining

Golgi-cox staining was performed with the FD Rapid GolgiStain™ kit from FD NeuroTechnologies (Columbia, MD, USA) according to the manufacturer’s instructions. Briefly, animals were sacrificed by cervical dislocation without anaesthesia, the brains rapidly dissected out and immerged in impregnation solution for 2 weeks at room temperature in the dark. Tissues were then transferred into solution C and incubated for 3 days in the dark. One-hundred micrometre thick sections were cut with a frozen cryostat, mounted on gelatine-coated slides (#PO101, FD NeuroTechnologies) and air-dried for 2 days in the dark. The slides were then washed two times 4 min in distilled water, incubated for 10 min in the staining solution, rinsed two times 4 min with water, dehydrated and coverslipped with Permount mounting medium (Thermo Fisher Scientific). Brightfield microscopy images of CA1 pyramidal dendrites were acquired using the MIRAX SCAN scanner (Zeiss) with an ×100 objective. For each mouse, a minimum of 15 neurons and 1–3 dendrites per neuron of 25–35 μm length was used for analysis. The analyzed dendritic areas belonged to neurons with visible soma, were located at least 25 μm from the soma and did not overlap with other cells. Results were expressed as total number of spines per 10 μm of uninterrupted section. Tissue processing and quantifications were blinded to genotype.

### Statistical analyses

Data are expressed as means ± SEM. Data were tested for equivalence of variance with Bartlett’s test. Comparison between two groups was done using the unpaired Student’s two-tailed *t*-test. Statistical analyzes of more than two groups were performed with one-way analysis of variance (one-way ANOVA) followed by the Bonferroni’s post-hoc test. For repeated measures, the two-way ANOVA with genotype as one factor and time for the other, followed by the Bonferroni’s post-hoc test was used. Statistical calculations were performed using GraphPad Prism 5.0 software (GraphPad, La Jolla, CA, USA). Results were considered significantly different at *p* < 0.05.

## Supplementary information

Supplementary materials

## Data Availability

The data generated and analyzed for this study are available from the corresponding author on reasonable request. Digital scans of immunohistological staining have been saved electronically and can be made available upon request and provision of a depository site with sufficient memory to accept the files. Any additional information is available upon request.
