# Supplementary material for: Caspase-6-cleaved Tau fails to induce Tau hyperphosphorylation and aggregation, neurodegeneration, glial inflammation, and cognitive deficits
Source: Cell Death Dis. 2021 Mar 1;12(3):227. doi: 10.1038/s41419-021-03506-0 (PMC7921451; doi:10.1038/s41419-021-03506-0)
Supplement: Supplementary file 1 — Supplementary materials [file 41419_2021_3506_MOESM1_ESM.docx]

***Supplementary materials for***

**Caspase-6-cleaved Tau fails to induce Tau hyperphosphorylation and aggregation, neurodegeneration, glial inflammation, and cognitive deficits.**

Anastasia Noël ^1,2^, Bénédicte Foveau ^1,2^, Andréa C. LeBlanc ^1,2,3^

^1^ Bloomfield Center for Research in Aging, Lady Davis Institute for Medical Research, Jewish General Hospital, 3755 Chemin Côte Ste Catherine, Montreal, QC, Canada H3T 1E2

^2^ Department of Neurology and Neurosurgery, McGill University, 3755 University Street, Montreal, Québec, QC, Canada H3A 2B4

^3^ Department of Anatomy and Cell Biology, McGill University, 3755 University Street, Montreal, Québec, QC, Canada H3A 2B4

**Running title:** Caspase-6-cleaved Tau is not toxic.

**Corresponding author:** Andréa LeBlanc, PhD, Bloomfield Center for Research in Aging, Lady Davis Institute for Medical Research, Jewish General Hospital, 3755 Chemin Côte Ste Catherine, Montreal, QC, Canada H3T 1E2. Tel.: +1 (514) 340 8222 ext 25303; e-mail: [andrea.leblanc@mcgill.ca](mailto:andrea.leblanc@mcgill.ca)

**Supplementary Table 1.** Fisher's exact test p values for the comparison of the NOR impaired CTC KI/Cre and CTO KI/Cre mice with their respective control genotypes at 3, 9, 18 and 25 months of age.

|  | ***p value*** | **CTC KI/Cre** | **CTO KI/Cre** | **CTC KI/WT** | **CTO KI/WT** | **WT/Cre** | **WT/WT** |
| --- | --- | --- | --- | --- | --- | --- | --- |
| **3M** | **CTC KI/Cre** |  | 1.0000 | 1.0000 |  | 0.3326 | 0.2241 |
|  | **CTO KI/Cre** | 1.0000 |  |  | 0.0897 | 0.3150 | 0.0996 |
| **9M** | **CTC KI/Cre** |  | 1.0000 | 0.1917 |  | 1.0000 | 1.0000 |
|  | **CTO KI/Cre** | 1.0000 |  |  | 1.0000 | 1.0000 | 1.0000 |
| **18M** | **CTC KI/Cre** |  | 1.0000 | 0.3333 |  | 0.4815 | 1.0000 |
|  | **CTO KI/Cre** | 1.0000 |  |  | 1.0000 | 1.0000 | 1.0000 |
| **25M** | **CTC KI/Cre** |  | 1.0000 | 1.0000 |  | 1.0000 | 1.0000 |
|  | **CTO KI/Cre** | 1.0000 |  |  | 1.0000 | 1.0000 | 0.6581 |

**Supplementary Table 2.** Fisher's exact test p values for the comparison of the Barnes maze impaired CTC KI/Cre and CTO KI/Cre mice with their respective control genotypes at 3, 9, 18 and 25 months of age.

|  | ***p value*** | **CTC KI/Cre** | **CTO KI/Cre** | **CTC KI/WT** | **CTO KI/WT** | **WT/Cre** | **WT/WT** |
| --- | --- | --- | --- | --- | --- | --- | --- |
| **3M** | **CTC KI/Cre** |  | 0.4828 | n.d. |  | 0.2258 | **0.0421*** |
|  | **CTO KI/Cre** | 0.4828 |  | 1.0000 |  | 1.0000 | 0.3898 |
| **9M** | **CTC KI/Cre** |  | 1.0000 | 0.6059 |  | 0.5996 | 0.5996 |
|  | **CTO KI/Cre** | 1.0000 |  |  | 1.0000 | 1.0000 | 0.5996 |
| **18M** | **CTC KI/Cre** |  | 0.3845 | 1.0000 |  | 1.0000 | 0.5645 |
|  | **CTO KI/Cre** | 0.3845 |  |  | 0.0671 | 0.3845 | 0.0671 |
| **25M** | **CTC KI/Cre** |  | 1.0000 | 0.5333 |  | 0.1029 | 1.0000 |
|  | **CTO KI/Cre** | 1.0000 |  |  | 0.1748 | **0.0473*** | 1.0000 |

*n.d.: not determined*

**
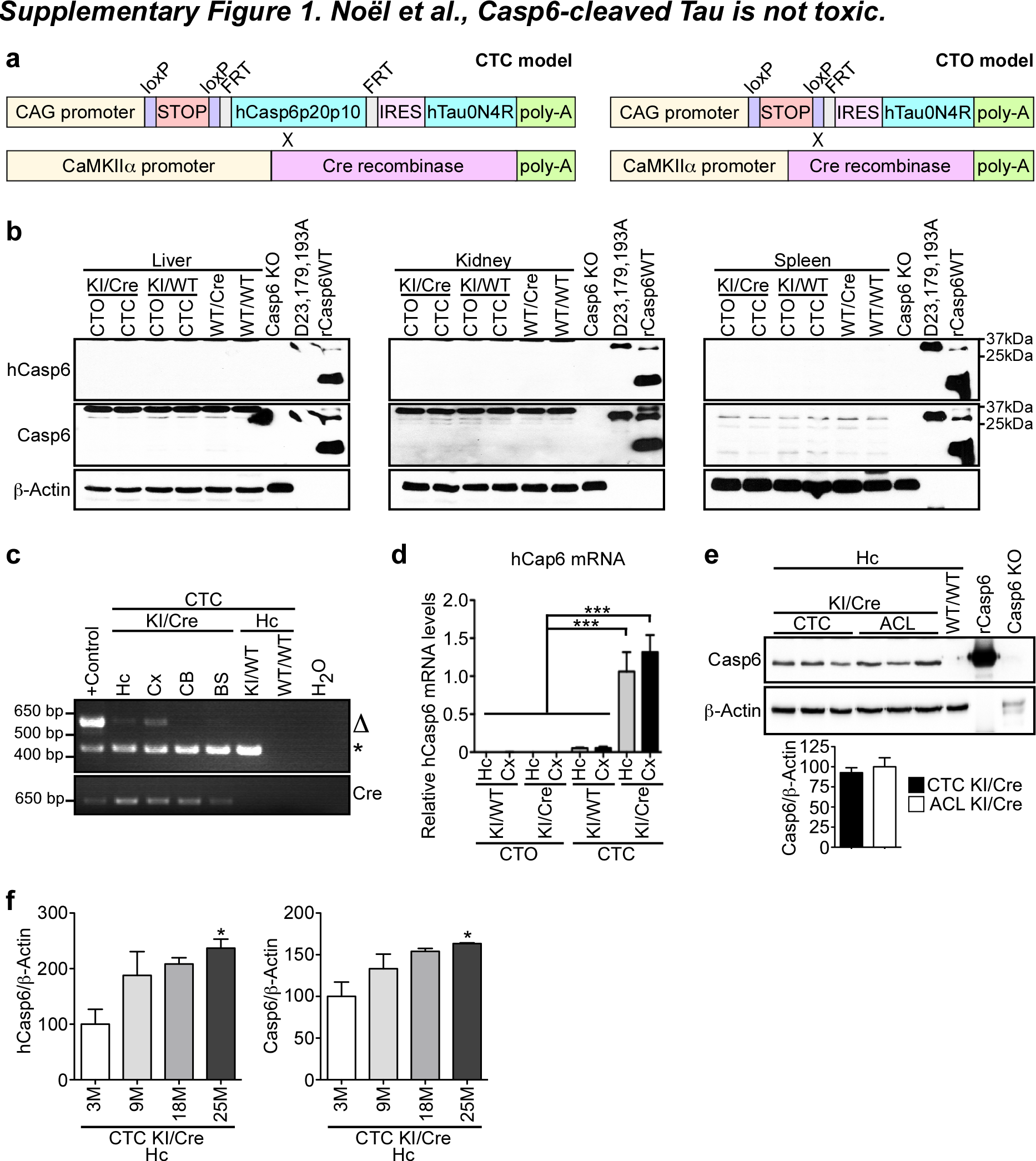
**

**Supplementary Figure 1. CTC KI/Cre mice express hCasp6 under the CaMKIIa-Cre action. (a)** Schematic diagram showing the constructs in transgenic mice crossed to generate the Conditional hTau-hCasp6 expressing (CTC) and Conditional hTau Only expressing (CTO) models. CAG promoter: ubiquitous cytomegalovirus immediate early enhancer fused to the chicken β-Actin (CAG) promoter, IRES: internal ribosomal entry site sequence, hCap6p20p10: hCasp6 cDNA lacking its pro-domain, FRT: Flippase recognition target sites. **(b)** Western blot analyses with anti-hCasp6, anti-total Casp6 and anti-β-Actin antibodies of 15 μg total proteins from 25-month-old CTO KI/Cre, CTC KI/Cre, CTO KI/WT, CTC KI/WT, WT/Cre and WT/WT liver, kidney and spleen. Casp6 KO protein extract was used as negative control. Recombinant unprocessed full-length Casp6 D23, 179, 193A (D23,179,193A) and Casp6WT were used as positive controls. **(c)** Red safe-stained agarose gel showing excision of the STOP sequence in the CTC KI/Cre mouse hippocampus and cortex by multiplex PCR amplification of the non-excised (*) and Cre-excised (Δ) knocked-in *Hprt* allele. Positive control for *Hprt* allele: mix of hCasp6-hTau transgene comprising the STOP cassette, with hCasp6-hTau transgene devoid of the STOP cassette. Genomic DNA from a T29-1 CaMKIIα-Cre mouse was used as positive control for the Cre gel. PCR without DNA (H_2_O) or with wild-type genomic DNA (WT/WT) were used as negative controls. Hc: hippocampus. **(d)** RT-qPCR analyzes of hCasp6 mRNA in the hippocampus (Hc) and cortex (Cx) of KI/WT and KI/Cre mice (n=3/structure/genotype, 2 months of age). One-way ANOVA followed by Bonferroni's post-hoc, ***p<0.001. **(e)** Western blot analyses with anti-total Casp6 and anti-β-Actin antibodies of 15 μg total proteins from 3-month-old CTC KI/Cre, ACL KI/Cre and WT/WT hippocampus (n=3/genotype). Unpaired Student’s two-tailed t-test (p=0.59). Casp6C163A (rCasp6) was used as positive control. **(f)** Quantifications of western blots shown in Figure 1c. Results are expressed as hCasp6 or Casp6 over β-Actin, relative to 3-month-old CTC KI/Cre hippocampus (n=3/age). One-way ANOVA followed by Bonferroni's post-hoc, *p<0.05 *vs* 3-month-old CTC KI/Cre.

**
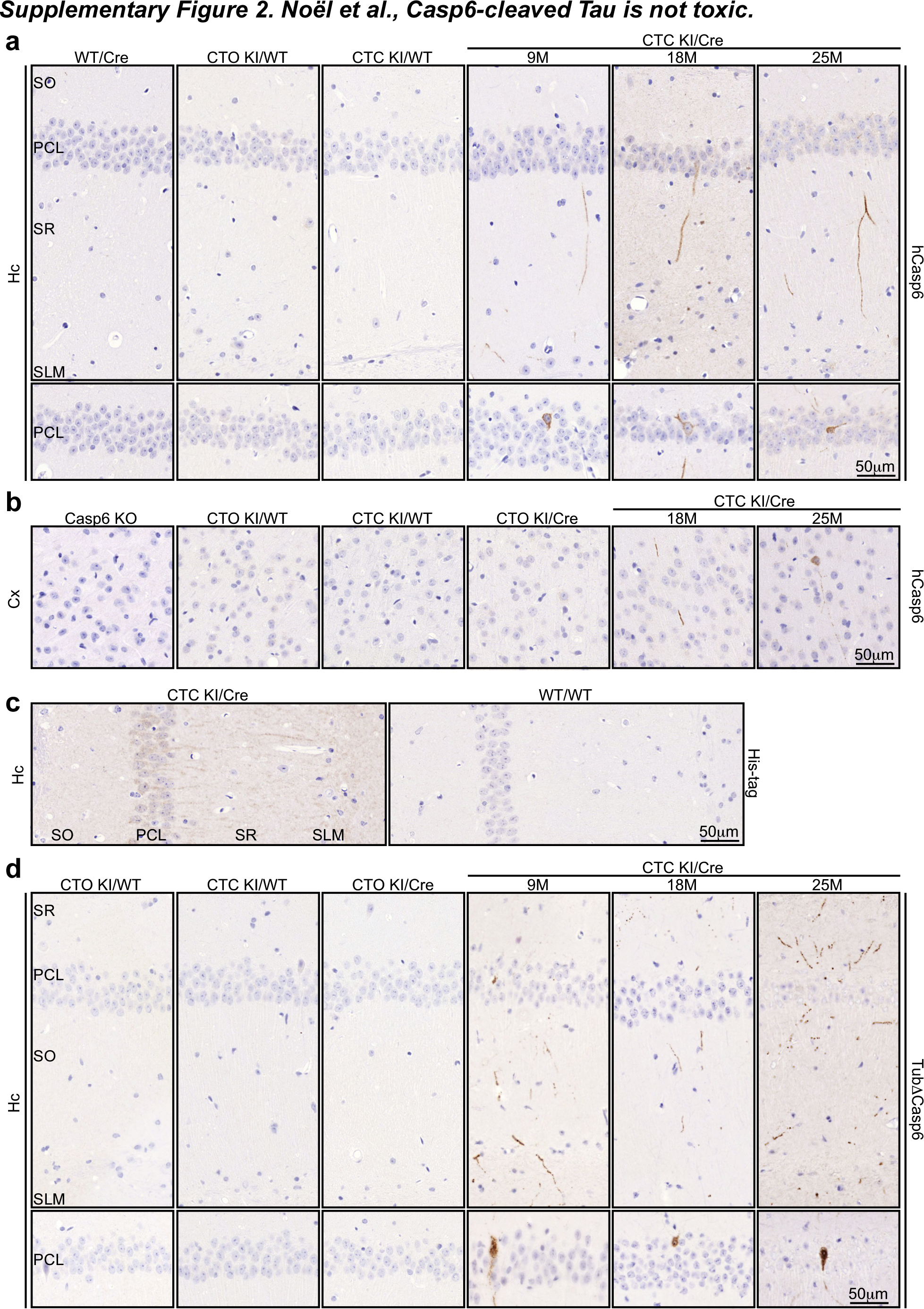
**

**Supplementary Figure 2. Specific expression of active hCasp6 in the hippocampus and cortex of CTC KI/Cre mice throughout aging.** Representative micrographs of (**a,c,d**) hippocampal or (**b**) cortical sections from WT/Cre, CTO KI/WT, CTC KI/WT, CTC KI/Cre and Casp6 KO mice stained with (**a-b**) anti-hCasp6, (**c**) anti-his-tag, or (**d**) anti-TubΔCasp6 antibody. n=3-5/genotype/age/structure. SO: stratum oriens, PCL: pyramidal cell layer, SR: stratum radiatum, SLM: stratum lacunosum molecular.


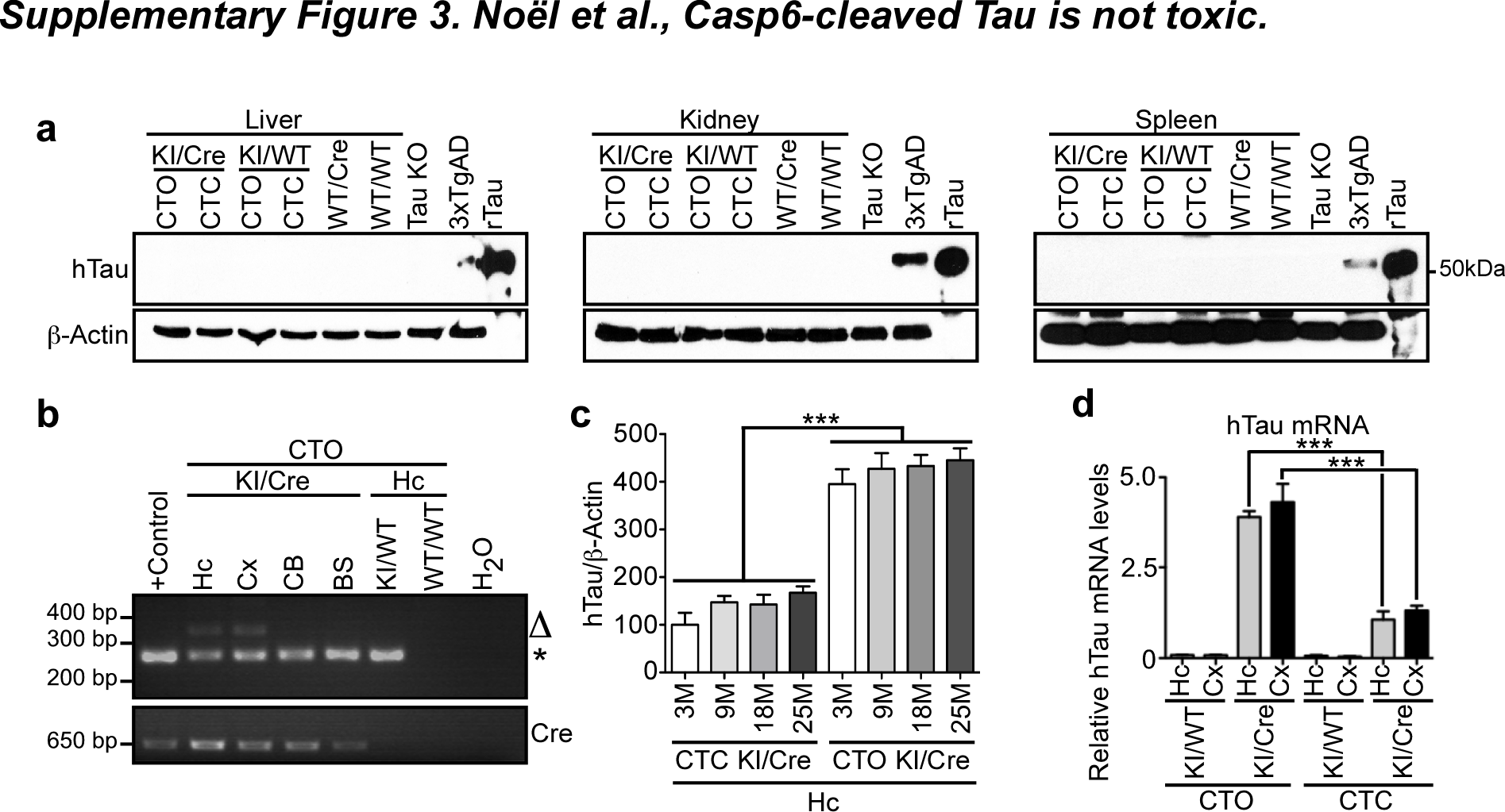


**Supplementary Figure 3. CTC and CTO KI/Cre mice express hTau under the CaMKIIa-Cre action. (a)** Western blot of 15 μg total proteins from 25-month-old CTO KI/Cre, CTC KI/Cre, CTO KI/WT, CTC KI/WT, WT/Cre and WT/WT liver, kidney and spleen with anti-hTau and anti-β-Actin antibodies. Lysates from Tau KO hippocampus was used as negative control. Protein extract from 3xTgAD and recombinant hTau were used as positive controls. **(b)** Red safe-stained agarose gel showing excision of the STOP sequence in the CTO KI/Cre mouse hippocampus and cortex by multiplex PCR amplification of the non-excised (*) and Cre-excised (Δ) knocked-in *Hprt* alleles. Positive control for non-excised knocked-in *Hprt* allele: hCasp6-hTau transgene comprising the STOP cassette. Genomic DNA from a T29-1 CaMKIIα-Cre mouse was used as positive control for the Cre gel. PCR without DNA (H_2_O) or with wild-type genomic DNA (WT/WT) were used as negative controls. Hc: hippocampus, Cx: cortex, CB: cerebellum, BS: brainstem. **(c)** Densitometric analyses of western blots shown in Figure 1c. Results are expressed as ratios of hTau over β-Actin, relative to 3-month-old CTC KI/Cre. **(d)** RT-qPCR analyzes of hTau mRNA in the hippocampus (Hc) and cortex (Cx) of 2-month-old KI/WT and KI/Cre mice. **(c&d)** n=3/genotype. One-way ANOVA followed by Bonferroni's post-hoc test, ***p<0.001.


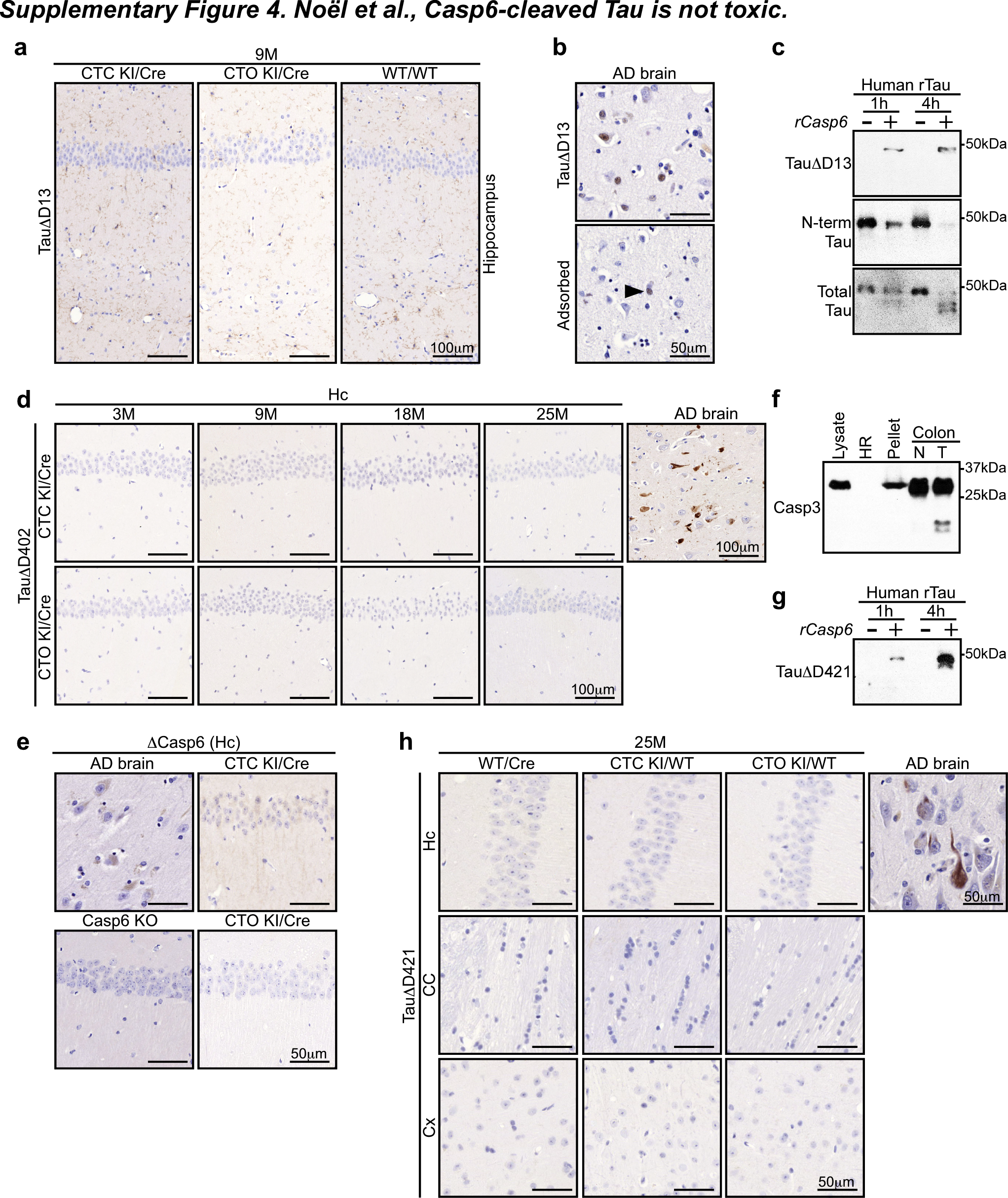


**Supplementary Figure 4. Casp6 directly cleaves Tau at D421 and TauΔD421 is present in the CTC KI/Cre brains. (a-b)** Representative micrographs of brain sections from (**a**) WT/WT, CTC and CTO KI/Cre, or (**b**) human AD stained with anti-TauΔD13 antibody. **(c)** Western blot analyses of human recombinant Tau0N4R (rTau) incubated with recombinant human active Casp6p20p10 (rCasp6) (+) or vehicle (-) for 1h or 4h with anti-TauΔD13, anti-hTau (N-term Tau) and anti-total Tau antibodies. **(d)** Representative micrographs of brain sections from 3- to 25-month-old CTC and CTO KI/Cre, or human AD stained with anti-TauΔD402. **(e)** Representative micrographs of brain sections from human AD, Casp6 KO, CTC and CTO KI/Cre stained with anti-processed Casp6 (ΔCasp6) antibody. **(f)** Western blot analyses of Casp3 in murine brain lysate, heat resistant (HR) and heat unstable proteins (pellet). Murine normal colon (N) or AOM-DSS induced colon tumor (T) lysates were used as controls for full length and processed Casp3. **(g)** Western blot analyses of human recombinant Tau0N4R (rTau) incubated with recombinant human active Casp6p20p10 (rCasp6) (+) or vehicle (-) for 1h or 4h with anti-TauΔD421 antibody. **(h)** Representative micrographs of brain sections from 25-month-old WT/Cre, CTC KI/WT and CTO KI/WT, or human AD stained with anti-TauΔD421 antibody.


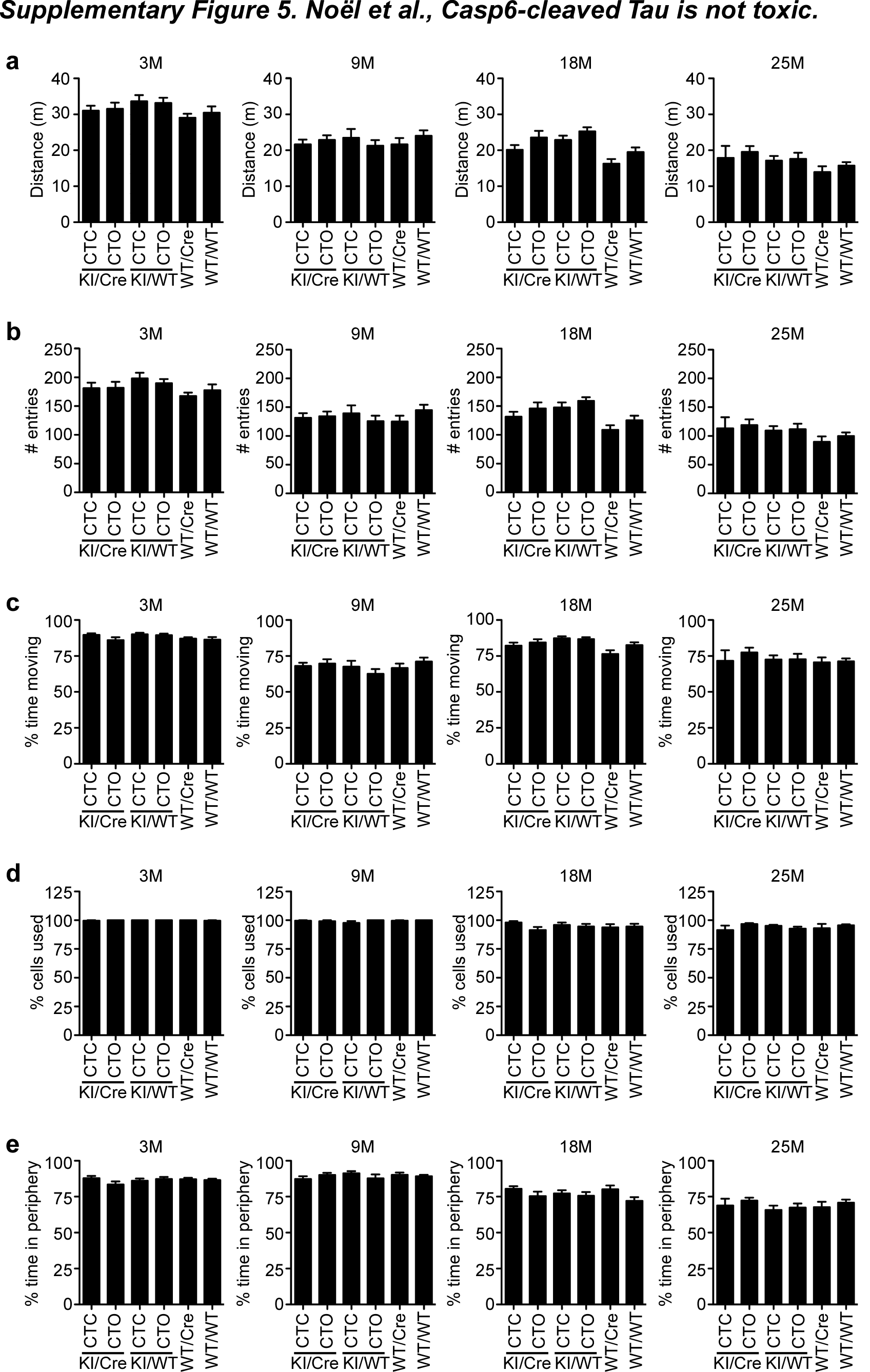


**Supplementary Figure 5. CTC and CTO KI/Cre mice perform normally during the openfield test. (a)** Total distance travelled, **(b)** total number of entries, **(c)** percentage of time moving, **(d)** percentage of cells used, and **(e)** percentage of time spent in the periphery of CTC KI/Cre (3M: n= 15, 9M: n=16, 18M: n=13, 25M: n=9), CTO KI/Cre (3M: n=14, 9M: n=15, 18M: n=14, 25M: n=17), CTC KI/WT (3M: n=15, 9M: n=13, 18M: n=15, 25M: n=14), CTO KI/WT (3M: n=16, 9M: n=15, 18M: n=17, 25M: n=16), WT/Cre (3M: n=16, 9M: n=15, 18M: n=14, 25M: n=10), and WT/WT (3M: n=15, 9M: n=16, 18M: n=17, 25M: n=18) at 3, 9, 18 and 25 months of age. Data represent the mean ± SEM.


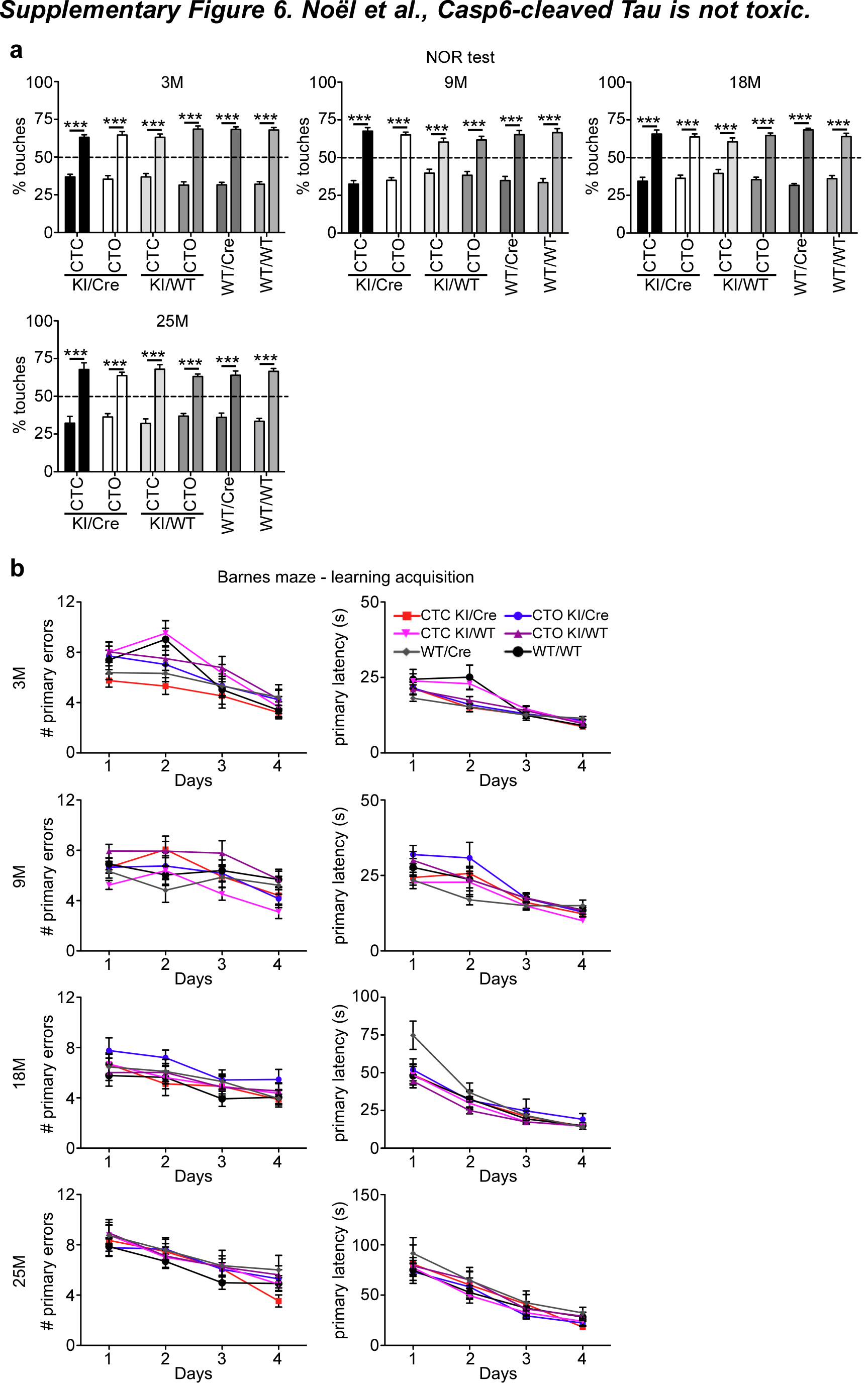


**Supplementary Figure 6. CTC and CTO KI/Cre mice perform normally during the cognitive tests. (a)** Percentage of touches to the familiar (left bars) and novel objects (right bars) during the novel object recognition test (NOR) and **(b)** number of primary errors and primary latency (s) to the target during the Barnes maze learning acquisition of 3 to 25-month-old CTC KI/Cre (3M: n= 15, 9M: n=16, 18M: n=13, 25M: n=9 for NOR and n=7 for Barnes maze), CTO KI/Cre (3M: n=14 for NOR and n=15 for Barnes maze, 9M: n=15, 18M: n=14, 25M: n=17), CTC KI/WT (3M: n=15, 9M: n=13, 18M: n=15, 25M: n=14), CTO KI/WT (3M: n=16, 9M: n=15, 18M: n=17, 25M: n=16), WT/Cre (3M: n=16, 9M: n=15, 18M: n=14, 25M: n=10), and WT/WT (3M: n=15 for NOR and n=14 for Barnes maze, 9M: n=16, 18M: n=17, 25M: n=18 for NOR and n=17 for Barnes maze). Statistical evaluations were done with (**a**) one-way ANOVA followed by a Bonferroni's post-hoc analysis, or (**b**) repeated-measures ANOVAs, with genotype and days as factors. Data represent the mean ± SEM. ***p<0.001.


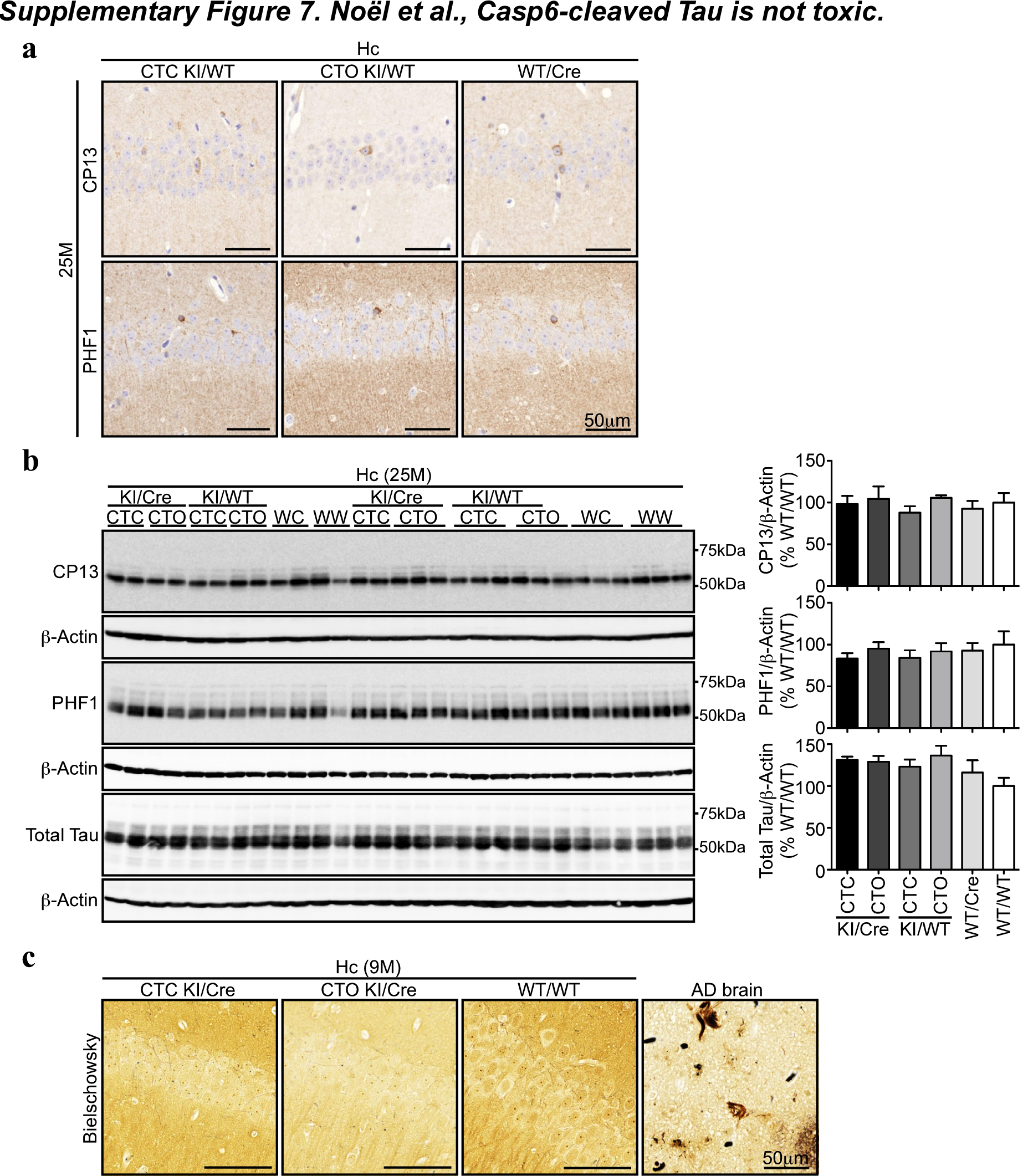


**Supplementary Figure 7. The levels of phosphorylated Tau are not increased in the hippocampus of old CTC KI/Cre. (a)** Representative micrographs of hippocampal sections from 25-month-old CTC KI/WT, CTO KI/WT and WT/Cre stained with CP13 or PHF1. (**b**) Western blot of 10 μg total hippocampal proteins from 25-month-old CTO and CTC KI/Cre, CTO and CTC KI/WT, WT/Cre and WT/WT with CP13, PHF1, anti-total Tau and anti-β-Actin antibodies. The histograms represent densitometric analyses of ratios of CP13, PHF1 or total Tau over β-Actin, relative to WT/WT (n=4-5/genotype). One-way ANOVA followed by Bonferroni's post-hoc test was performed. *p<0.05. **(c)** Representative micrographs of Bielschowsky silver staining of 9-month-old CTC and CTO KI/Cre, or WT/WT hippocampus tissue sections. Human AD brain was used as positive control. Hc: hippocampus.


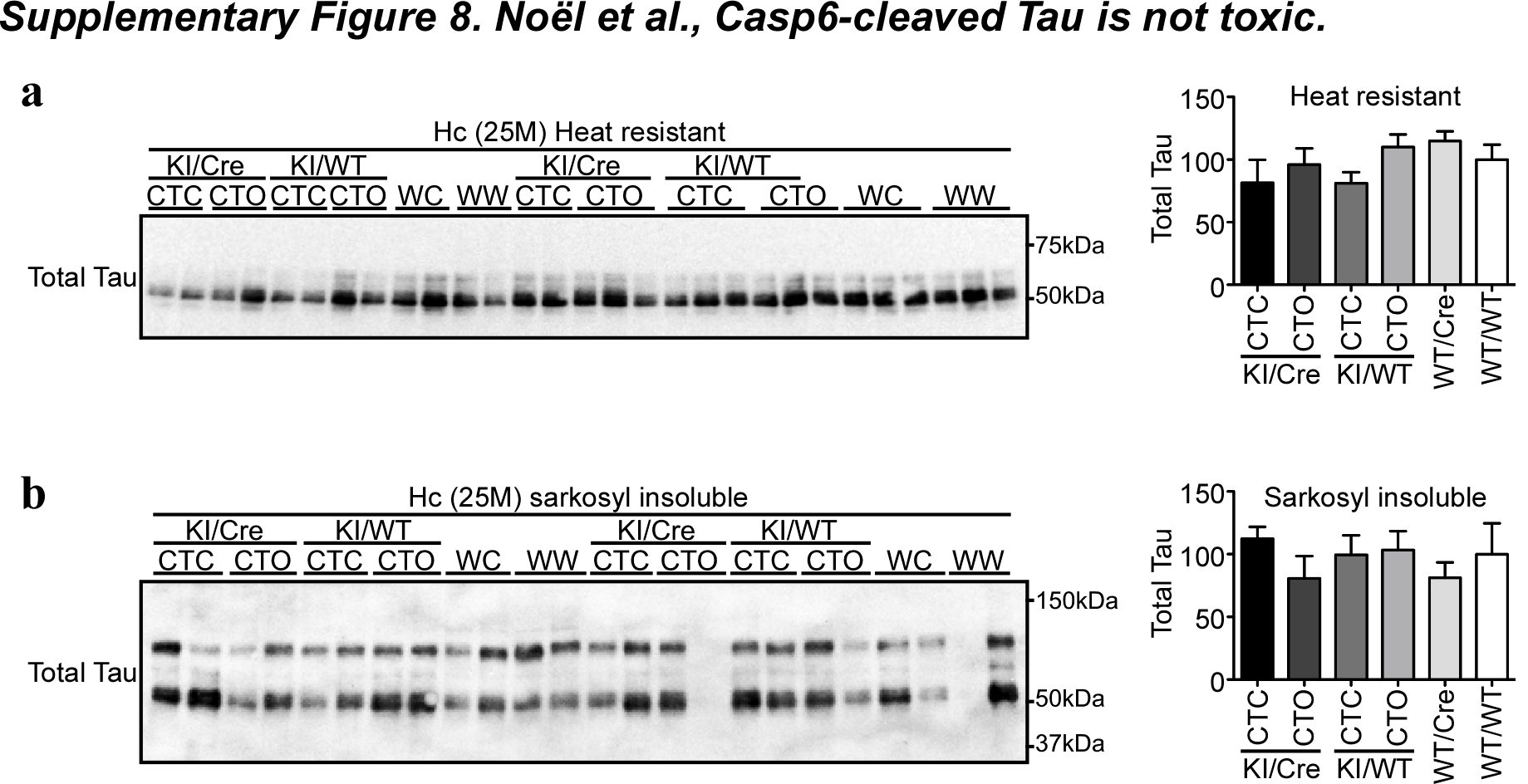


**Supplementary Figure 8. Sarkosyl insoluble Tau is not significantly increased in the hippocampus of old CTC KI/Cre.** Western blot analysis of total Tau in **(a)** the heat resistant protein hippocampal fraction or **(b)** the sarkosyl insoluble protein hippocampal fraction from 25-month-old CTC and CTO KI/Cre, CTC and CTO KI/WT, WT/Cre and WT/WT (n=4-5/genotype). Data represent the mean ± SEM. One-way ANOVA followed by Bonferroni's post-hoc test. Hc: hippocampus, WC: WT/Cre, WW: WT/WT.
